# Novel multiparameter correlates of *Coxiella burnetii* infection and vaccination identified by longitudinal deep immune profiling

**DOI:** 10.1038/s41598-020-69327-x

**Published:** 2020-08-07

**Authors:** P. M. Reeves, S. Raju Paul, L. Baeten, S. E. Korek, Y. Yi, J. Hess, D. Sobell, A. Scholzen, A. Garritsen, A. S. De Groot, L. Moise, T. Brauns, R. Bowen, A. E. Sluder, M. C. Poznansky

**Affiliations:** 1 Vaccine and Immunotherapy Center, Massachusetts General Hospital, Boston, MA USA; 2 Colorado State University, Fort Collins, CO USA; 3 InnatOss Laboratories B.V, Oss, The Netherlands; 4 EpiVax, Inc, Providence, RI USA; 5 Center for Vaccines and Immunology, University of Georgia, Athens, GA USA; 6 Institute for Immunology and Informatics, Department of Cell and Molecular Biology, University of Rhode Island, Providence, RI USA

**Keywords:** Immunology, Adaptive immunity, Vaccines, Microbiology, Pathogens, Vaccines

## Abstract

Q-fever is a flu-like illness caused by *Coxiella burnetii* (*Cb*), a highly infectious intracellular bacterium. There is an unmet need for a safe and effective vaccine for Q-fever. Correlates of immune protection to *Cb* infection are limited. We proposed that analysis by longitudinal high dimensional immune (HDI) profiling using mass cytometry combined with other measures of vaccination and protection could be used to identify novel correlates of effective vaccination and control of *Cb* infection. Using a vaccine-challenge model in HLA-DR transgenic mice, we demonstrated significant alterations in circulating T-cell and innate immune populations that distinguished vaccinated from naïve mice within 10 days, and persisted until at least 35 days post-vaccination. Following challenge, vaccinated mice exhibited reduced bacterial burden and splenomegaly, along with distinct effector T-cell and monocyte profiles. Correlation of HDI data to serological and pathological measurements was performed. Our data indicate a Th1-biased response to *Cb*, consistent with previous reports, and identify Ly6C, CD73, and T-bet expression in T-cell, NK-cell, and monocytic populations as distinguishing features between vaccinated and naïve mice. This study refines the understanding of the integrated immune response to *Cb* vaccine and challenge, which can inform the assessment of candidate vaccines for *Cb*.

## Introduction

Development of a safe, non-reactogenic and effective vaccine for Q-fever, a zoonotic disease found worldwide caused by the obligate intracellular bacteria *Coxiella burnetii* (*Cb*), represents a current unmet need. Approximately 40% of *Cb*-infected individuals experience flu-like symptoms, while a subset develop pneumonia, endocarditis and/or hepatitis^1–4^. *Cb* can persist in the environment for long periods and inhalation of < 20 bacteria can cause infection, raising public health concerns^5–9^. The current Q-fever vaccine for humans, Q-VAX, utilizes inactivated whole-cell virulent *Cb* (phase I Henzerling strain) to elicit protective immunity against *Cb*^10–12^. Q-VAX requires pre-screening to avoid reactogenicity in previously *Cb*-exposed individuals and is not considered suitable for mass vaccination^2,8,10,13–16^. Subunit vaccines containing *Cb* epitopes to elicit protective T-cell responses are a proposed strategy to bypass concerns related to LPS-induced reactogenicity^17–20^, while pre-clinical evaluation of candidate vaccines bearing computationally identified human-specific epitopes can be accomplished in mice expressing human MHC alleles^21–23^. The objective of this study was to generate immune profiling data using mass cytometry, along with serological and pathological assessments, to identify novel correlates of effective vaccination and control of *Cb* infection that could ultimately inform the development of a safe and effective vaccine for Q-fever.

*Cb*-infected individuals may retain epitope specific T-cell responses and anti-*Cb* antibodies for > 8 years, though up to 20% become seronegative 4–6 years following infection^24,25^. Immunologic studies in mice demonstrate that MHC-II dependent responses are required for effective vaccination and T-cells predominantly act to limit disease severity and burden, while B and NK cell responses contribute to clearance^26–29^. To further investigate the immune response to *Cb*, we sought to quantify the broader immune response to *Cb* in a vaccine–challenge model in mice. We conducted a longitudinal assessment of cellular and humoral immune responses to vaccination in transgenic mice expressing the human MHC-II allele HLA-DR3 on a BL/6 background (tgHLA-DR3)^30^. Vaccination with Coxevac, a veterinary vaccine containing inactivated whole-cell virulent *Cb,* was followed by challenge with the same strain of *Cb* (phase-I Nine Mile strain)^31^. Mass cytometry (CyTOF) was used to provide a comprehensive description of all major immune populations following vaccination and infection, and multivariate statistical methods were used to evaluate the correlation of cell populations to antibody generation, histopathology, and bacterial load. We identified novel correlates of *Cb* vaccination and infection characterized by expression of Ly6C, CD73, and T-bet, among other key markers across distinct T-cell, B-cell, and innate populations, and observed that key features of this response are detected in vaccinated mice. Our results reveal the dynamic and broad immune response to *Cb* to support the development of subunit-based vaccines for *Cb* and inform future investigations into immune pathogenesis of this and other intracellular pathogens.

## Results

### Determination of the vaccine dose that confers protection against *Cb* infection

BL/6 mice, the tgHLA-DR3 background strain, were injected with increasing doses of Coxevac and intranasally (i.n.) challenged with *Cb* 42 days post-vaccination (Supplementary Fig. 1A)^26^. Ten days after challenge, mice were sacrificed to quantify splenic bacterial burden and splenomegaly, and to conduct histopathological scoring of heart, lung, liver, and spleen (Supplementary Fig. 1). Increasing doses of Coxevac progressively reduced measures of infection. Vaccination with 2 μg was sufficient to reduce splenomegaly, as measured by spleen-to-body-weight ratio (%BW) and histopathological scoring, though not splenic burden (Supplementary Fig. 1B–D). Vaccination with 10 μg effectively reduced all measures of infection and was used for subsequent experiments.

### Longitudinal immunological assessment of *Cb* vaccination and challenge

**Figure 1:**
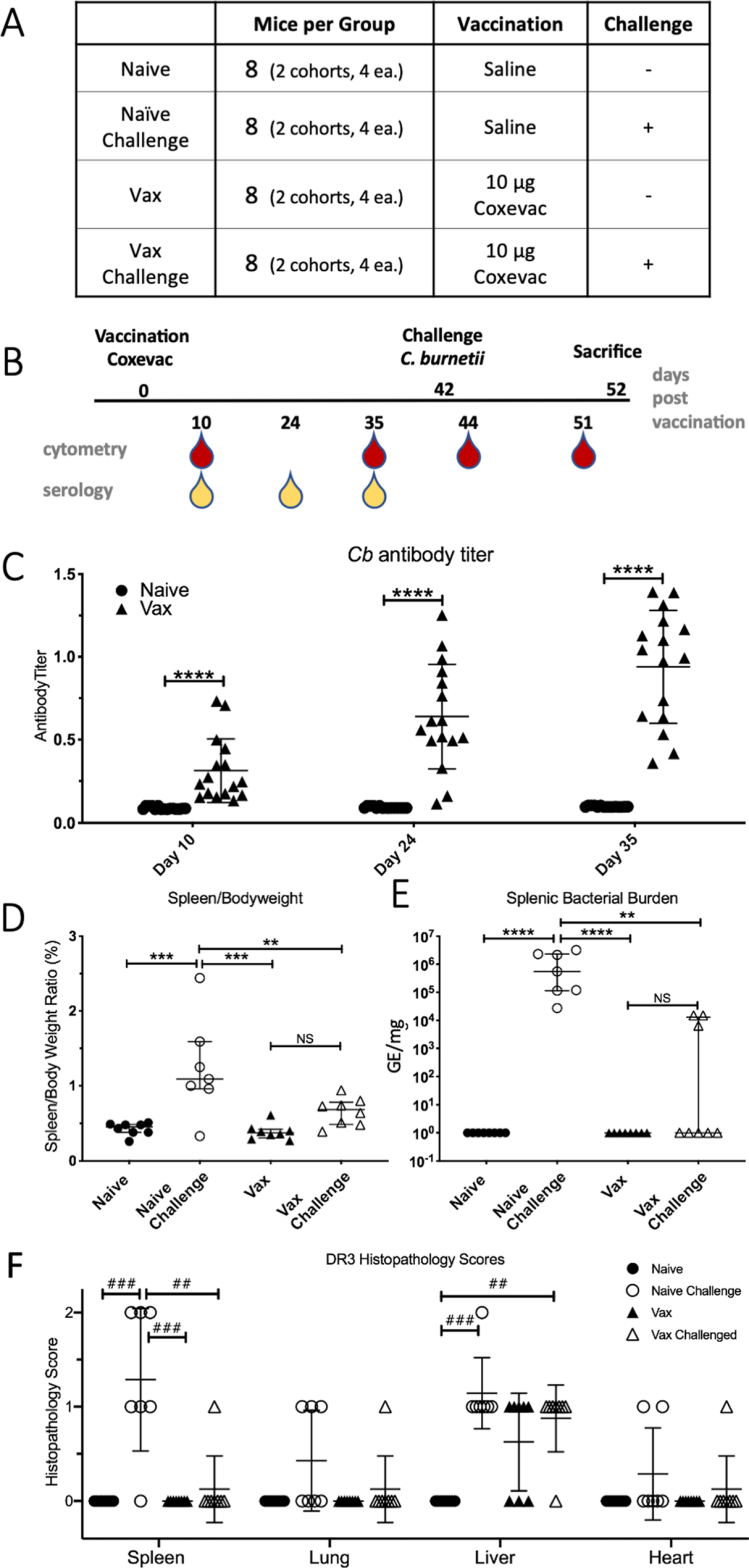
Clinical outcomes of Coxevac vaccination and *Cb* challenge in tgHLA-DR3 mice. (**A**) Treatment groups and numbers of mice for the tgHLA-DR3 study (**B**) Experimental schedule. Mice were injected subcutaneously with saline or 10 µg Coxevac on day 0. After 42 days mice were challenged intranasally with live *Cb*. Whole blood was collected for CyTOF analysis on Day 10, 35, 44 and 51. Sera were collected for serology on Day 10, 24 and 35. (**C**) Antibody production against *Cb* was evaluated at Day 10, 24, and 35 post-vaccination by ELISA (**D**) Spleen-to-body-weight ratio and (**E**) spleen bacterial burden (genome equivalents (GE) determined by qPCR) were assessed for each of the experimental groups. Significant differences between experimental groups in panels (**C**–**E**) were assessed by one-way ANOVA with the Tukey post-hoc multiple comparison correction (****p = 0.0001, ***p < 0.0003, *p < 0.01). (**F**) Histopathological scores from lung, liver, spleen, and heart. Kruskal–Wallis test was used to compare between groups (^###^p = 0.0003, ^##^p = 0.005).

We assessed the longitudinal profile of cellular immune responses to vaccination and challenge in tgHLA-DR3 mice in two independent replicate studies (Fig. 1A). Each study included 16 mice divided into naïve and vaccinated groups (n = 8 per group per study) that were sub-divided into challenge and uninfected groups (n = 4 per group per study, Fig. 1A). One mouse assigned to the naïve-challenge group died on day 35, prior to challenge. On day 42 post-vaccination, a subset of naïve and vaccinated mice was challenged i.n. with *Cb*. Blood was collected on days 10, 24, and 35 post-vaccination and days 2 and 10 post-infection (days 44 and 51 post-vaccination) (Fig. 1B). For each cytometry timepoint, surface antigens were antibody-labelled following collection, and samples fixed to inactivate viable *Cb* (Supplementary Table 1). Following confirmation of inactivation and release from biocontainment, intracellular epitopes were labeled, and samples analyzed by mass cytometry.

Pre-challenge antibody titers and quantitative measures of infection were also determined. *Cb*-specific antibodies increased from Days 10 to 35 post-vaccination (Fig. 1C). Following sacrifice on Day 10 post-challenge, measures of bacterial burden, splenomegaly, and histopathology were determined. Challenge with *Cb* significantly increased splenomegaly (%BW) and bacterial burden in naïve mice as compared to unchallenged mice (either naïve or vaccinated). In vaccinated mice, challenge did not significantly increase splenic measures of *Cb* burden or %BW, as compared to unchallenged mice (Fig. 1D–E). Vaccination similarly reduced histology scores for spleen, heart and lung. However, both vaccination and challenge independently resulted in elevated liver histopathology (Fig. 1F). For vaccinated challenged mice, all but one exhibited moderate hepatic histopathology (score = 1). One vaccinated challenged mouse had elevated spleen %BW, heart and spleen histopathology, though no bacterial burden. Among the three vaccinated challenged mice with detectable bacterial burden, only one was found to have both lung and spleen histopathology. Thus, vaccination with inactivated whole-cell *Cb* elicited protective immunity sufficient to reduce bacterial burden and pathological measures overall, with evidence of vaccine enhancement of splenic clearance by 10 days post-challenge in a subset of mice.

### Identification of immune cell populations consistent between replicate experiments

The high-parameter nature of mass cytometry data can provide multi-marker definitions of immune populations for a detailed description of immune responses. Computational tools can simultaneously assess the abundance of markers on individual cells to identify clusters of highly similar cells. We used a barcoding strategy to antibody label and acquire all samples from each timepoint as one cohesive unit. However, technical variations between timepoints and between replicate experiments resulted in batch effects that precluded simultaneous identification of populations from all timepoints and replicates by clustering analysis.

We developed an approach to combine data from the two replicate experiments and thereby improve statistical power for comparisons among treatment groups (Supplementary Fig. 2). Briefly, each timepoint from individual replicate experiments was analyzed independently and cell clusters conserved between replicate timepoints were identified (e.g. Day 10 experiment 1 and Day 10 experiment 2). First, cell profiling data from each timepoint was analyzed individually to identify cell clusters computationally using X-shift^32^. Next, the resulting clusters from replicate timepoints were matched on the basis of their phenotypic profiles and candidate pairs refined according to abundance and overall distribution between treatment groups. For the resulting “matched clusters” representing clusters conserved between two replicate timepoints, the data were combined to improve statistical power of comparisons among treatment groups (see “Methods”).

### Temporal changes in abundance of phenotypic immune cell classes

**Figure 2:**
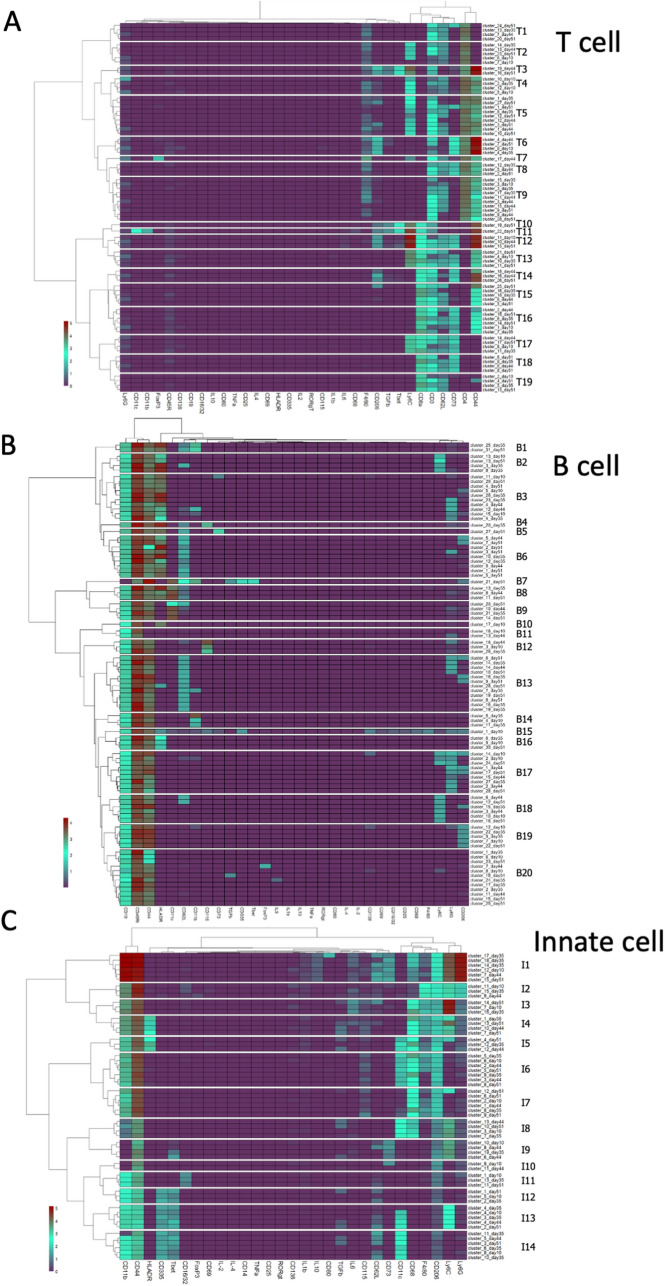
Identification of phenotypic classes of immune cell populations. Matched cell clusters from all timepoints, determined as described in the text, were organized hierarchically (Y axes) based on relative marker expression levels (X axes and heatmap color coding), using the Ward.D2 minimum variance method. Similar clusters were grouped into phenotypic classes (delineated by white boundaries in heat maps); the number of classes were selected from unsupervised analysis using NbClust and segmentation determined using dendrogram tree height. Hierarchical maps are shown for T cell (**A**), B Cell (**B**), and innate immune populations (**C**).

Matched clusters from all timepoints were organized hierarchically to identify phenotypically similar clusters from across the experiment for T-cell, B-cell, and innate populations (Fig. 2). The x-axis dendrogram indicates the influence of each marker in defining cell classes. The antibody panel, which recognized a broad range of immune populations, provided subsets of markers that dominate the heatmap for each of the three major populations. Through hierarchical clustering, we identified “phenotypic classes” of cells which contain matched clusters bearing similar, though not identical, marker profiles. Some classes contain clusters from multiple days or multiple clusters from a single day. Phenotypic classes containing a single matched cluster were included, as they represented features observed in two independent experiments. The analysis of T-cells resulted in 77 matched clusters in 19 phenotypic classes (Fig. 2A). For B-cells, 92 matched clusters separated into 20 phenotypic classes (Fig. 2B). Within innate immune populations, 60 matched clusters segregated into 14 phenotypic classes (Fig. 2C).

**Figure 3:**
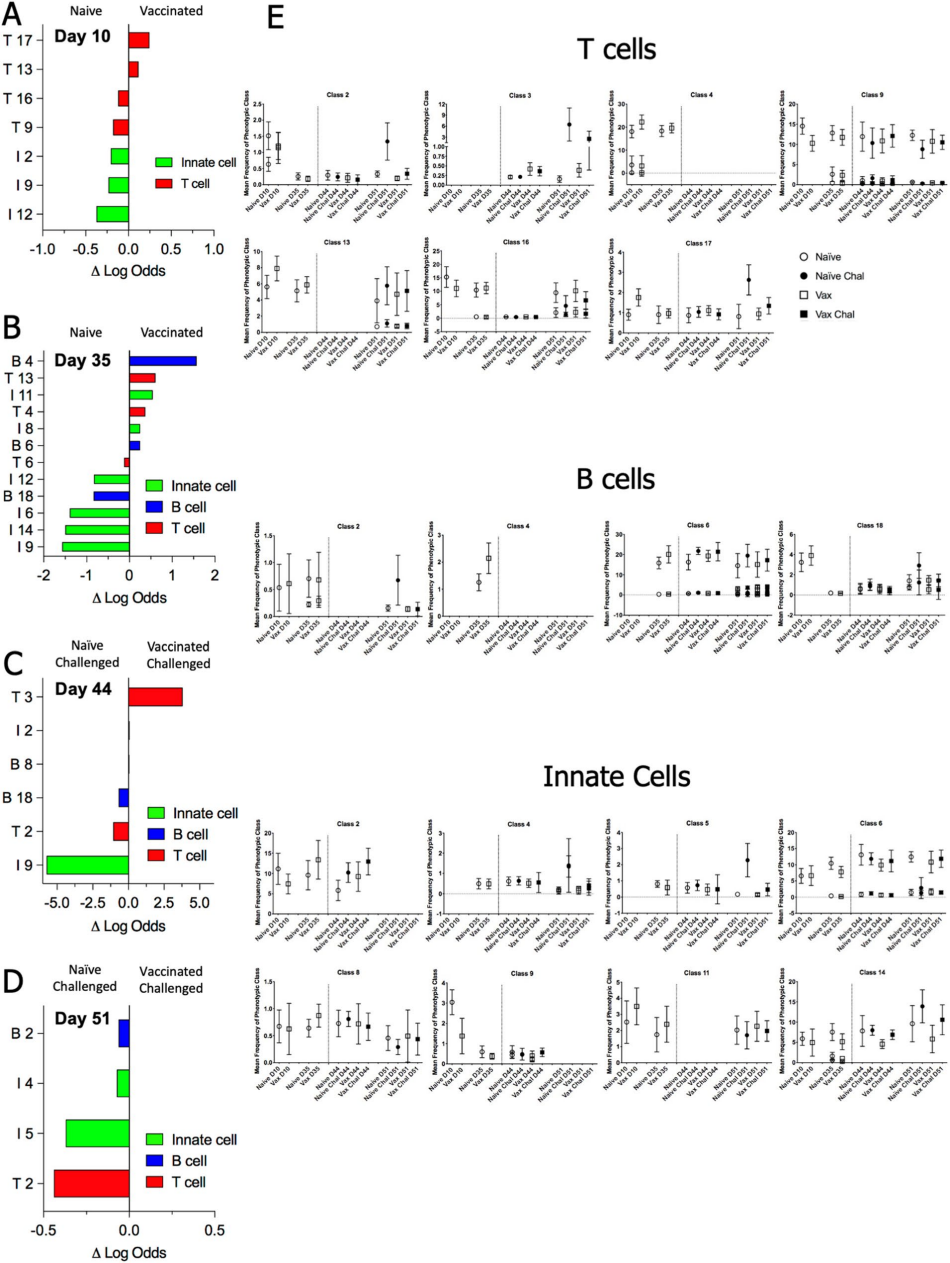
Distribution of phenotypic classes across vaccination and challenge. For each timepoint, an EN regression analysis was used to determine the probability of assignment to either experimental group, using the median frequency of each phenotypic class per mouse on that day. The graphs identify cell classes with highest predictive value for distinguishing vaccinated vs naïve mice pre-challenge (**A**,**B**) or vaccinated challenged v naïve challenged mice (**C**,**D**) based on the magnitude of their logit coefficient, which is a measure of the influence of change in the abundance of the cluster between experimental groups on the predictive value of the cluster. Phenotypic class names are as assigned in Fig. 2. In panel E, plots indicate the median frequency and standard deviation of a phenotypic class per experimental group across timepoints for selected classes that have high predictive value. The dotted vertical line indicates intranasal challenge with *Cb*. For phenotypic classes with multiple clusters at a given timepoint, all clusters are plotted individually. Plots of all classes are shown in Supp Fig. 4A–C are provided in supplementary data.

The capacity of each phenotypic class to predict classification of an individual mouse as naïve or vaccinated was determined for pre-challenge timepoints using an elastic net (EN) logistic regression analysis (Fig. 3A,B)^33^. EN analysis was conducted on post-challenge data to identify those classes that distinguish vaccinated challenged and naïve challenged mice (Fig. 3C,D). To evaluate the temporal distribution of key classes, the frequency of each phenotypic class through the time course was determined (Fig. 3E, Supplementary Fig. 4A–C). Following vaccination, on Day 10 T-cell and innate immune classes distinguish naïve and vaccinated groups, with activated T-cells linked to vaccinated mice. After 35 days, predictive B-cell classes appear, with Day 10 T-cell and innate cell trends continuing. Challenge strongly polarized the predictive immune populations within 2 days post-challenge. By Day 51, the most predictive clusters were activated CD4+ T-cells and monocyte/macrophages found in naïve challenged mice. Detailed observations related to phenotypic families, as well as subsequent evaluation of individual clusters and correlation to experimental outcomes, are summarized in Table 1 and described further in the Supplementary Material.

**Table 1:** Summary of cell populations associated with vaccination status.

Phenotypic class (Fig. 2)		EN Clusters (Fig. 5)	Correlation Module (Fig. 4)	Key Markers Expressed
ID	EN Timepoint (Fig. 3)			
T2	D10 Vaccinated D51 Naïve Challenged	T D10 Cluster 6 T D44 Cluster 13 T D51 Cluster 23	3 5 4	CD4 CD62L Ly6C
T3	D44 Vaccinated Challenged	T D44 Cluster 19	1	CD4 CD44 Ly6C T-bet TGFb
T4	D35 Vaccinated	T D10 Cluster 5 T D35 Cluster 2	3 6	CD4 CD62L CD44 Ly6C
T9	D10 Vaccinated	T D10 Cluster 3 T D35 Cluster 15 T D35 Cluster 3	5 7 5	CD4 CD44 CD62L
T13	D10 Vaccinated D35 Vaccinated	T D10 Cluster 4 T D35 Cluster 10	1 1	CD8 CD62L CD44 Ly6C CD73
T16	D10 Naïve D35 Vaccinated	T D10 Cluster 1 T D35 Cluster 6	4 6	CD8 CD62L CD44 CD73
T17	D10 Vaccinated	T D10 Cluster 8 T D35 Cluster 11	1 7	CD8 CD62L Ly6C CD73
B2	D51 Naïve challenged	B D35 Cluster 3 B D51 Cluster 13	3 3	CD19 CD45R CD44 HLA-DR Ly6C
B4	D35 Vaccinated	B D35 Cluster 20	1	CD19 CD45R CD44 C62L HLA-DR CD115
B6	D35 Vaccinated	B D35 Cluster 10 B D35 Cluster 12	3 6	CD19 CD45R CD44 C62L HLA-DR
B18	D35 Naïve D44 Naïve challenged	B D35 Cluster 15	4	CD19 CD45R CD44 Ly6C
I2	D10 Naïve	I D10 Cluster 11	5	CD11b CD44 F4/80 CD206 Ly6C Ly6G
I4	D51 Naïve Challenged	I D51 Cluster 13	4	CD11b CD44 CD68 F4/80 CD206 Ly6C HLA-DR
I5	D51 Naïve Challenged	I D35 Cluster 12 I D51 Cluster 4	6 4	CD11b CD11c CD206 CD44 HLA-DR
I6	D35 Naïve	I D35 Cluster 5 I D35 Cluster 9 I D51 Cluster 5	4 7 1	CD11b CD11c CD206 CD44
I8	D35 Vaccinated	I D35 Cluster 17	2	CD11c CD44 CD68 CD206 Ly6C
I9	D10 Naïve D35 Naïve D44 Naïve Challenged	I D10 Cluster 10 I D35 Cluster 19 I D44 Cluster 6 I D44 Cluster 8	4 4 5 7	CD44 CD73 CD206 Ly6C
I11	D10 Vaccinated D35 Vaccinated	I D10 Cluster 1 I D35 Cluster 13	3 3	CD11b CD44 CD206 CD16
I12	D10 Naïve D35 Naïve	I D10 Cluster 5 I D35 Cluster 2	5 5	CD335 CD44 CD11b CD206 T-bet
I14	D35 Naïve	I D35 Cluster 6 I D35 Cluster 10 I D35 Cluster 11	5 4 5	CD335 CD44 CD11b CD206 T-bet CD11c

The table provides the information on cell phenotypic classes and clusters identified through statistical analyses as associated with either naïve or vaccinated mice pre- and post -challenge. For phenotypic classes selected by elastic net regression (EN), the class identity (ID, Fig. 2) and timepoint(s) are noted (Fig. 3). Within each phenotypic class, those matched clusters also identified by EN are noted, along with the correlation modules for each cluster (Supp Fig. 5).

### Correlation of cell clusters to measures of vaccination and infection

Grouping cell clusters into phenotypic classes provides information on the distribution of cell populations through time and enables detection of phenotypic cell classes with predictive value. However, this approach does not assess the relationship of individual populations to either experimental group or to measures of vaccination and infection. To determine the link of individual populations to measured physiological data and treatment group, we conducted correlation and EN logistic regression analyses.

**Figure 4:**
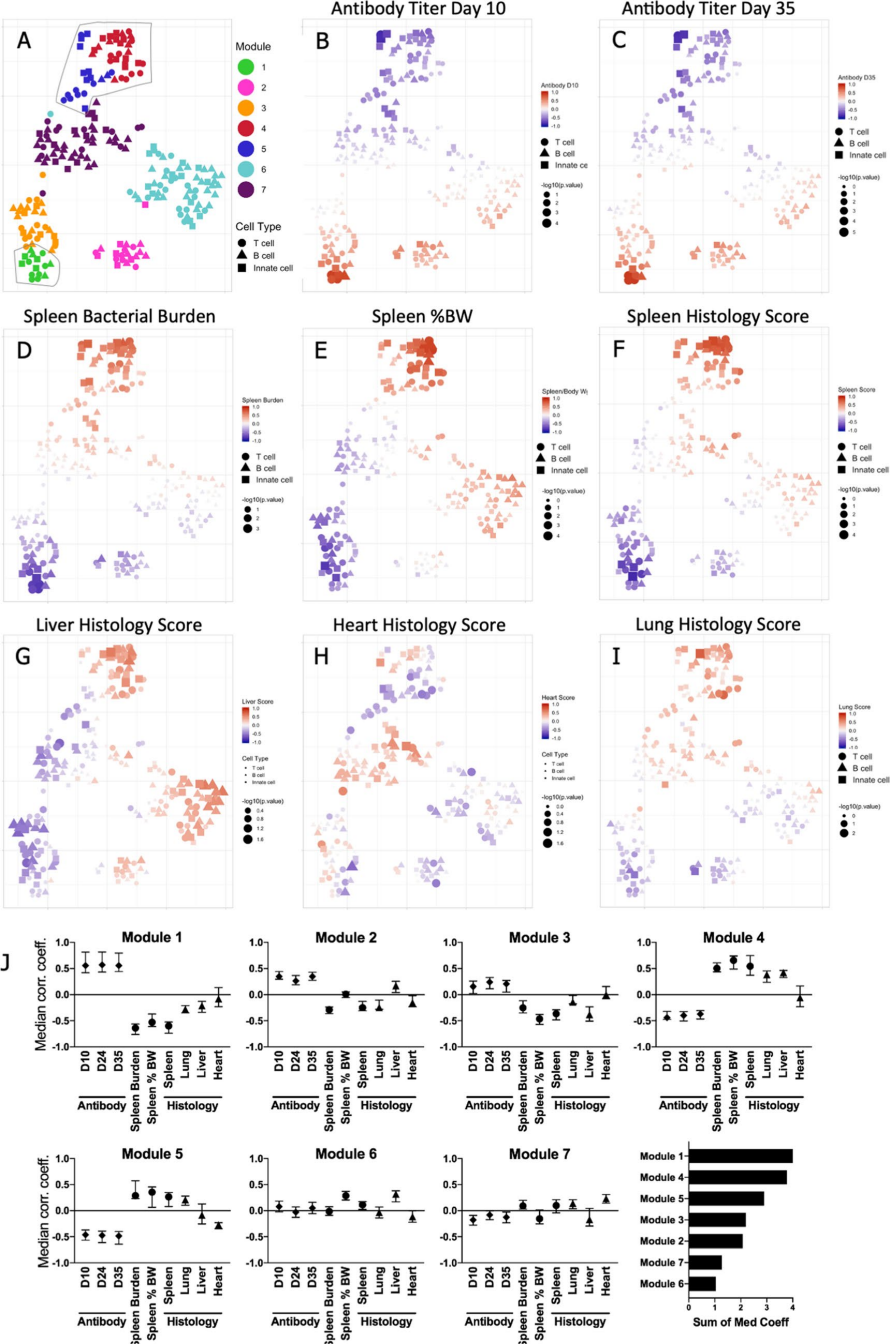
Correlation of clusters to measures of vaccination and infection. For matched clusters from tgHLA-DR3 mice, the correlation to measures of vaccination (antibody titer) and infection (spleen bacterial burden, spleen %BW, and histology scores) was determined (Supp Fig. 5). Correlation matrix data were used to generate a t-SNE map. T cell, B cell, and innate clusters are denoted by distinct shapes. (**A**) The hierarchical structure of the correlation matrix was used to identify 7 modules (Suppl Fig. 5), and each module was color-coded to indicate its position on the t-SNE. The outlined areas indicate the modules with the highest median correlation coefficient for clinical measures within the matrix (Module 1 lower left, Modules 4 and 5 upper right). (**B**–**I**) For each cluster, symbol size indicates correlation p-value while color is function of the correlation coefficient to the antibody titer on Day 10 and 35 post-vaccination (**B**,**C**), or measures of infection (**D**–**I**). (**J**) Individual panels show the median correlation coefficient for each clinical measure for each module. In the lower right graph, modules are ranked based on the sum of the median correlation coefficients.

First, we determined the correlation of each matched cluster to antibody titer, splenic %BW, bacterial burden, and histopathological scoring. Hierarchically organizing clusters on the basis of correlation values, independent of timepoint or cellular marker profiles, delineated seven modules which were visualized using a t-SNE plot^34^ (Fig. 4A, Supplementary Fig. 5, Supplementary Table 2). The spatial distribution of clusters within the t-SNE is a function of the similarity between cell clusters with respect to their correlations to measures of vaccination and infection (Fig. 4B–I). The subset of clusters in Module 1 (Supplementary Table 3) consistently correlated with elevated antibody titers (Fig. 4B,C; Day 24 titer not shown).

For each module, the median correlation of constituent clusters to antibody titer and measures of infection was determined (Fig. 4J, Supplementary Table 3). Module 1 had the strongest association with antibody titer and a negative correlation to all measures of infection. Modules 2 and 3 also correlated to increased antibody titer, though with elevated correlation to splenic measures, Module 2 also showed an increased correlation to liver histopathology, as compared with 1 & 3. Module 4 followed by Module 5 was the most linked to elevated histopathological scores and negatively correlated to antibody titer; their adjacent positioning on the t-SNE is consistent with their similar correlation properties. Overall, Modules 1, 4, and 5 had the strongest correlations to vaccination-induced antibody production or measures of infection. The module assignments for key populations are noted in Table 1.

### Predictive cells clusters to distinguish vaccinated from naïve mice

**Figure 5:**
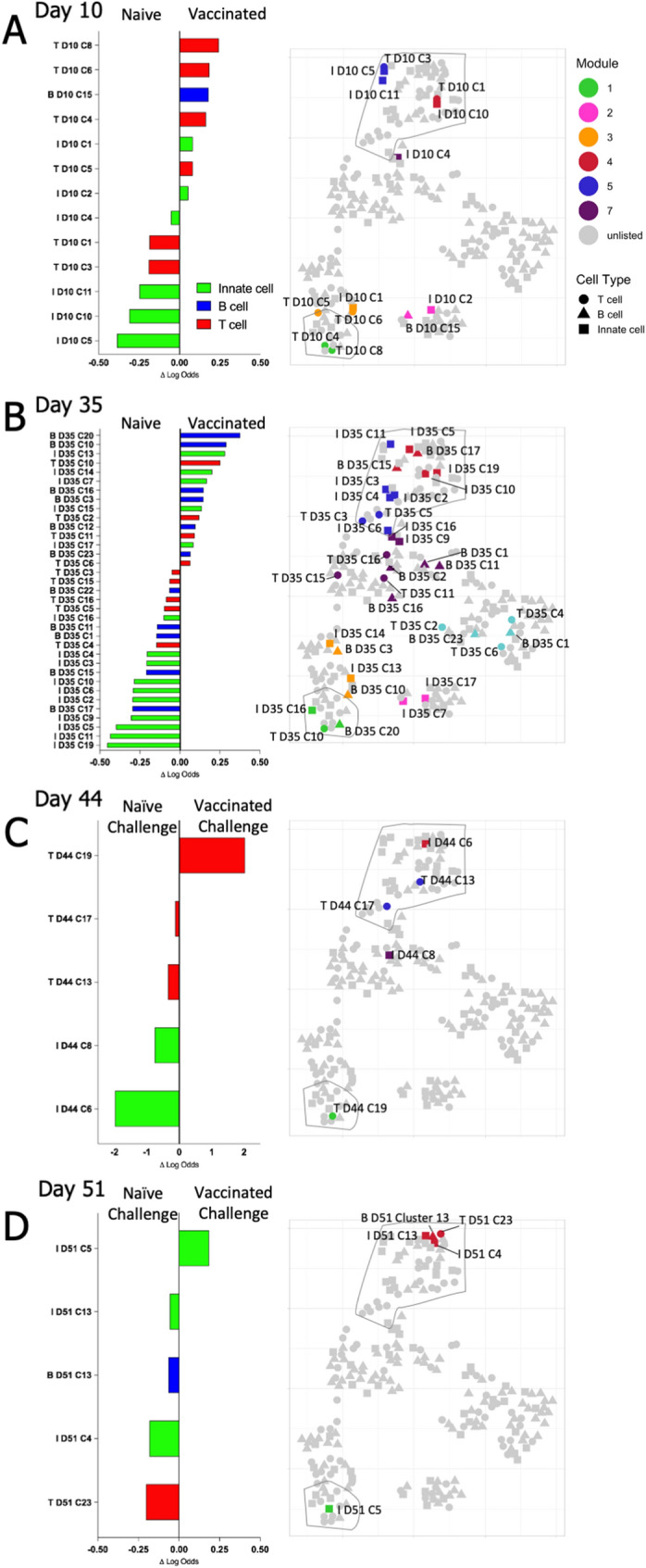
Matched Clusters Predictive of Experimental Treatment Group. For each timepoint, EN analysis identified matched clusters predictive of experimental groups. Clusters that distinguished (**A**,**B**) vaccinated from naïve mice, and (**C**,**D**) vaccinated challenged from naïve challenged mice, are plotted according to the magnitude of their logit coefficient, a measure of the influence of change in the abundance of the cluster between experimental groups on the predictive value of the cluster. The position of individual clusters identified by EN are indicated on the adjacent t-SNE map, coloring and outlined areas indicate modules defined in Fig. 4 (Module 1 lower left, Modules 4 and 5 upper right).

We next identified individual clusters and combinations of clusters that best differentiated, at each timepoint, between either vaccinated and naïve mice or between challenged naïve and vaccinated mice at each timepoint. Individual clusters were assessed as predictors of group assignment by EN logistic regression. The predictive clusters for each timepoint are graphed to show their relative rank, as well as position on the correlation module t-SNE map (Fig. 5A–D); individual predictive clusters are described further in the Supplementary Material. On Day 10, activated T-cell and B-cell responses distinguish vaccinated mice, while naïve mice exhibited resting T-cell and mature innate cell populations. By Day 35 a greater number of predictive populations were identified, with B-cell and innate populations most clearly distinguishing vaccinated mice, while mature monocyte and mature NK populations characterized naïve mice. Following challenge, the number of predictive clusters decreased, with T-cell and innate cell populations most prominently distinguishing challenged naïve and vaccinated groups.

To investigate the immune response in a more integrated manner, combinations of clusters that most clearly distinguished vaccinated from naïve mice following either vaccination or challenge with *Cb* were determined. The predictive capacity for every possible combination of up to 10 clusters for each timepoint was determined using four computational selection methods (Supplementary Fig. 3). The optimal combination of clusters for each timepoint was selected based on the consensus rank among the four measures, with strong agreement across methods regarding accuracy and sensitivity (Table 2). Notably, for each day,  the selected combination of clusters was ranked highest by the neural net (NN) method at all timepoints, other than Day 51, when a requirement that one or more clusters linked to vaccinated mice were included meant the combination ranked second by NN was chosen, wherein innate cluster 4 was exchanged for innate cluster 5 (Supplementary Table 3). Consistent with the individual cluster EN analysis, post-vaccination timepoints required more clusters to achieve maximum performance as compared to timepoints post-challenge: six clusters representing all major immune cell populations were required 10 days after vaccination to best distinguish vaccinated and naïve mice. By Day 35 post-vaccination, the combination of a single B-cell population and four innate immune populations, without T-cell populations, was sufficient to discriminate between vaccinated and naïve mice. Following challenge, vaccinated mice could be distinguished from naïve mice with four populations at two days post-challenge, and with two populations on Day 10 after challenge. At both post-challenge timepoints, the indicated T-cell and innate populations (Table 2) were sufficient to distinguish these groups, without requiring consideration of B-cell populations.

**Table 2:** Cluster sets to distinguish vaccinated and naïve mice after vaccination and challenge.

	Minimal cluster set distinguishing vaccinated and naïve mice following vaccination and challenge			
	D10	D35	D44	D51
Predictors	T D10 Cluster 6	B D35 Cluster 10	T D44 Cluster 19	I D51 Cluster 5
	B D10 Cluster 15	I D35 Cluster 6	T D44 Cluster 13	T D51 Cluster 23
	T D10 Cluster 1	I D35 Cluster 9	I D44 Cluster 8	
	I D10 Cluster 11	I D35 Cluster 11	I D44 Cluster 6	
	I D10 Cluster 10	I D35 Cluster 19		
	I D10 Cluster 5			
Accuracy	0.991267354	0.978123601	0.950747569	0.867281627
95% CI (Accuracy)	0.989597557–0.991618568	0.976273511–0.979696496	0.946311676–0.954665136	0.857867203–0.876290465
F1 Score	0.990884218	0.978223994	0.942810354	0.853730454
Sensitivity	0.990993093	0.989126377	0.927925458	0.838666939
Specificity	0.991520317	0.967975207	0.970250839	0.891832809

The table lists the minimal set of clusters that best distinguishes naïve and vaccinated mice on Day 10 and Day 35 or naïve challenged mice from vaccinated challenged mice on Day 44 and Day 51, determined using unsupervised computational analysis. The accuracy, 95% confidence interval (CI), F1 score, sensitivity, and specificity scores are listed for each time point. Further details are provided in Supplementary Table 3.

Together, these alterations in distribution of phenotypic classes and their constituent clusters provide immunological signatures that distinguish treatment groups (Table 1). Vaccination increased the abundance of CD8+ Ly6C+ CD73+ T-cells as well as CD4 + Ly6C+ T-cells by Day 10 post-vaccination. By Day 35 post-vaccination these CD4+ cells were not apparent, while CD8+ cells were also expressing CD44+, suggesting transition to a central memory phenotype. Both CD4+ and CD8+ T-cell populations expressing CD62L and Ly6C were substantially increased 10 days post-challenge. Post-challenge, these increases were more prominent in naïve mice, indicating an overall greater response from activated naïve T-cells.

These observations indicate that Ly6C+ cells in both naïve and memory peripheral T-cell populations are increased following exposure to *Cb*, either through vaccination or intranasal challenge. Notably, the majority of differences between treatment groups observed in innate cell populations were higher frequencies of circulating monocyte, macrophage, and NK cell populations in naïve mice compared to vaccinated mice, following either vaccination or challenge. In particular, T-bet+ NK populations, likely representing mature cells, were reduced following vaccination, suggesting their mobilization to sites of inoculation. Overall, the number, distribution, and respective phenotypes of clusters that best distinguish groups of mice at each timepoint (Table 2) identify hallmarks of the immunological processes induced by vaccination and the differential response to challenge in naïve and vaccinated mice.

**Figure 6:**
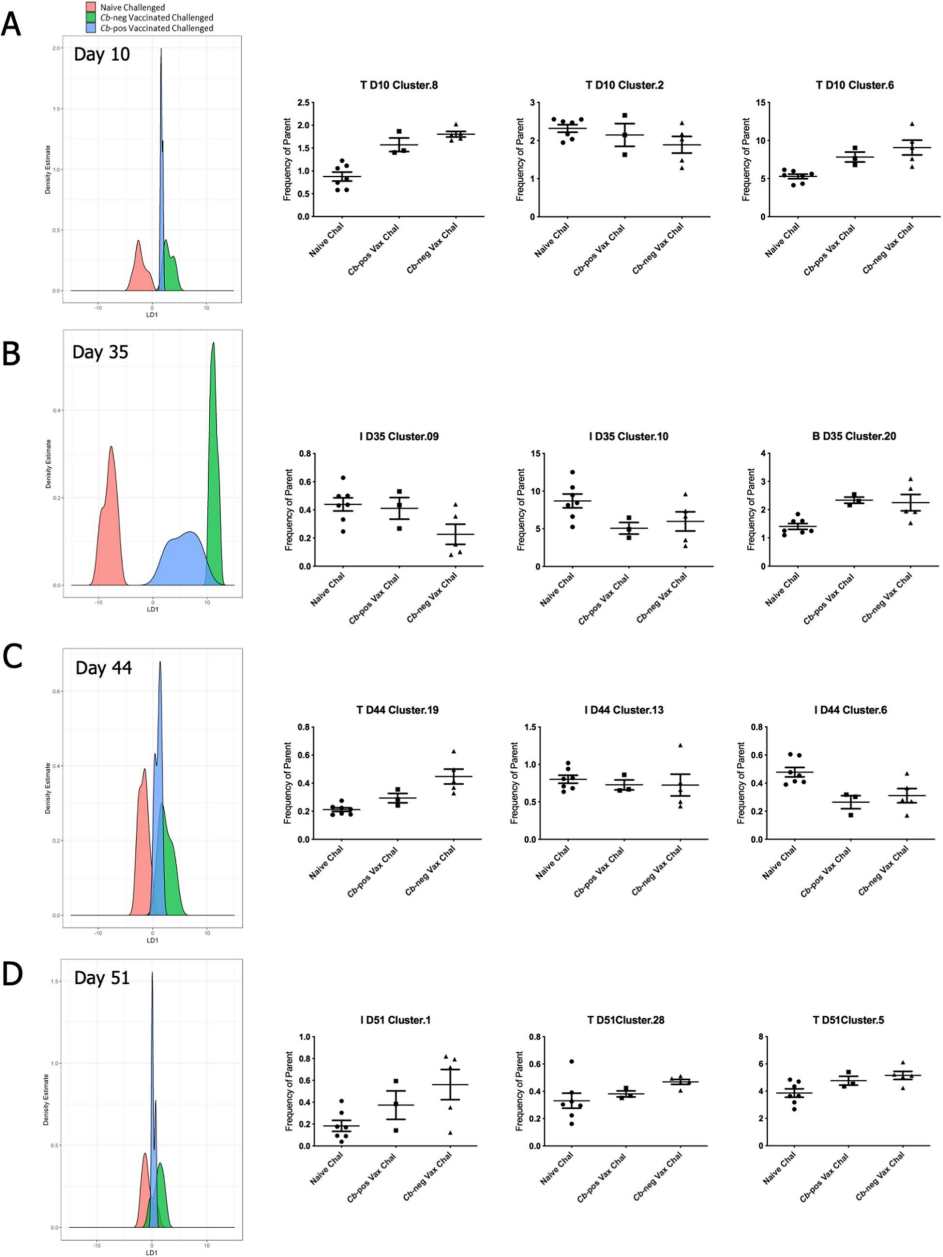
Identification of features characterizing vaccine efficacy following challenge. Linear discriminant analysis (LDA) models were constructed for each timepoint to rank combinations of up to 3 clusters based on the ability to distinguish naïve challenged mice (N = 7) from *Cb*-neg vaccinated challenged mice (N = 5) and subsequently assess the distribution of *Cb*-pos mice (N = 3). The discriminant functions with high predictive accuracy were used to project the three groups onto corresponding feature space and identify combinations of cell populations where the *Cb*-pos mice trend towards the naïve vaccinated mice. LDA plots depict the distribution of each group, where the center of the x-axis denotes the point where the naïve challenged and Cb-neg vaccinated challenged groups are maximally separated and the y-axis reflects the count (or density estimate). Adjacent to each LDA plot is a scatter plot for the respective constituents. The title of each plot indicates the cluster identity and the frequency parent on the y-axis.

We sought to determine if there were immunologic differences between the vaccinated mice that had no detectable bacterial burden by Day 10 post-challenge and the three vaccinated mice that retained detectable *Cb* (Fig. 1E). For each timepoint, combinations of up to three clusters were ranked by linear discriminant analysis (LDA) for ability to distinguish between naïve challenged mice and the *Cb*-negative vaccinated challenged mice. Next, to identify cell populations that distinguish *Cb*-positive vaccinated mice, the distributions of cluster combinations were compared between the three challenge groups: naïve, *Cb*-negative, and *Cb*-positive. On Day 10 post-vaccination three T-cell clusters distinguished challenged naïve and vaccinated mice and also showed a distribution in the three *Cb*-positive mice that trended towards naïve challenged mice (Fig. 6A). These three clusters correspond to naïve Ly6C+ CD4+ and CD8+ populations increased in vaccinated mice and a naïve Ly6C- CD8+ population enriched in naïve mice. Comparison of the three groups of mice on Day 35 identified a combination of mature NK cells and a monocyte population associated with naïve mice and an activated memory B-cell population in vaccinated mice (Fig. 6B). At two days post-challenge, a combination of CD4+ Ly6C+ Tbet+ T-cells and CD68+ mononuclear cells, both expressing TGFβ in vaccinated mice, along with a macrophage population in naïve challenged mice, was selected by the analysis (Fig. 6C). At Day 10 post-challenge distinctions between the three groups were less robust. Nonetheless, a combination of central memory CD8+, effector memory CD4+ T-cells, and a mature NK population all enriched in vaccinated mice was identified (Fig. 6D). Despite the small group size, these data suggest signatures associated with vaccine efficacy that can be further investigated in studies that include larger groups of mice or for evaluation of candidate *Cb* vaccines.

### Manual validation of marker expression in major immune cell populations

**Figure 7:**
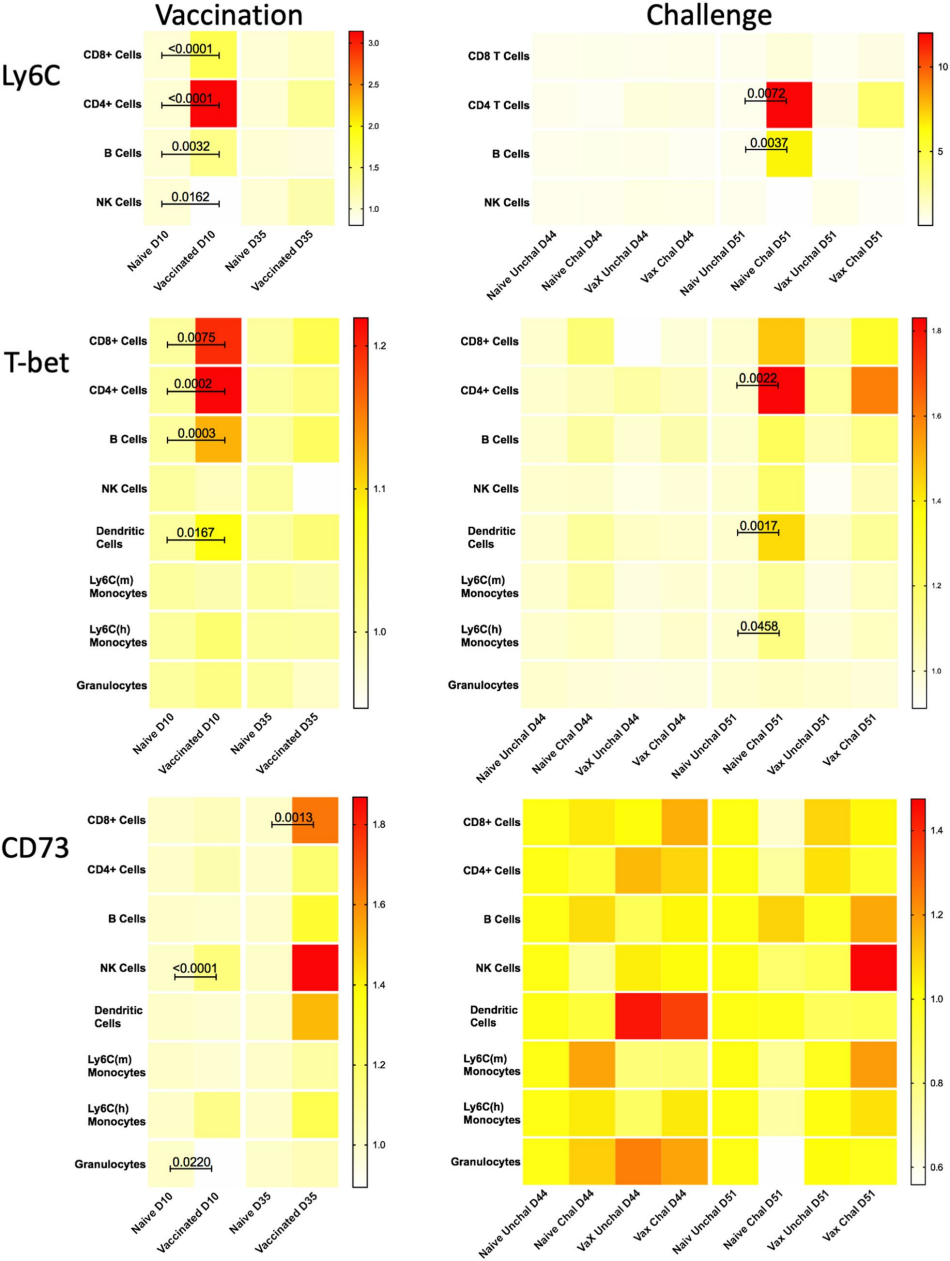
Manual assessment of expression of key markers following *Cb* vaccination and challenge. Manually gated populations were used to determine the frequency of parent for Ly6C, T-bet+, or CD73+ cells in major immune populations from tgHLA-DR3 mice. Heatmaps illustrate the median frequency of parent within each group of mice (n = 8) on Days 10 and 35 post-vaccination, and Days 2 and 10 post-challenge (study Days 44 and 51). The color-coded scale reflects the frequency of parent for each heatmap. The Kruskal–Wallis test for non-parametric data was used to identify statistically significant changes based on pairwise comparisons between treatment groups within each timepoint; the p-value for significant changes are indicated on the graph.

To confirm key features identified by the statistical analysis of computationally-defined cell populations, all major immune populations were manually gated (Supp Fig. 3B), and the frequency of cells expressing Ly6C, T-bet, or CD73 was determined. In this analysis, we observed increased expression of each of these individual markers following vaccination and challenge (Fig. 7), consistent with the changes in cellular phenotypes identified computationally (Table 1). Significantly increased expression of T-bet was seen 10 days post-vaccination in CD4+ T-cells and B-cells, and to a lesser, yet statistically significant, extent in CD8+ T-cells and dendritic cells. On Day 35 post-vaccination, the expression of T-bet had returned to near baseline levels in these cell populations. Ten days post-challenge, naïve mice exhibit higher levels of T-bet in CD4+ T-cells, dendritic cells, and to a lesser extent in inflammatory monocytes (Ly6C^high^ monocytes) as compared to vaccinated mice. The most pronounced increases in Ly6C expression were observed 10 days following vaccination in CD8+ and CD4+ T-cells. B-cells and NK cells also exhibited smaller, yet significant, post-vaccination increases in Ly6C expression at this timepoint. Two days after challenge, CD4+ T-cells and B-cells from naïve challenged mice exhibited a greater increase in Ly6C as compared to vaccinated challenged animals. CD73 expression increased in NK cells and to a lesser extent in granulocytes 10 days post-vaccination, and with statistical significance in CD8+ T-cells 35 days post vaccination. Thus, manual gating analysis confirms the key features and markers identified based on computational clustering of immune populations.

**Figure 8:**
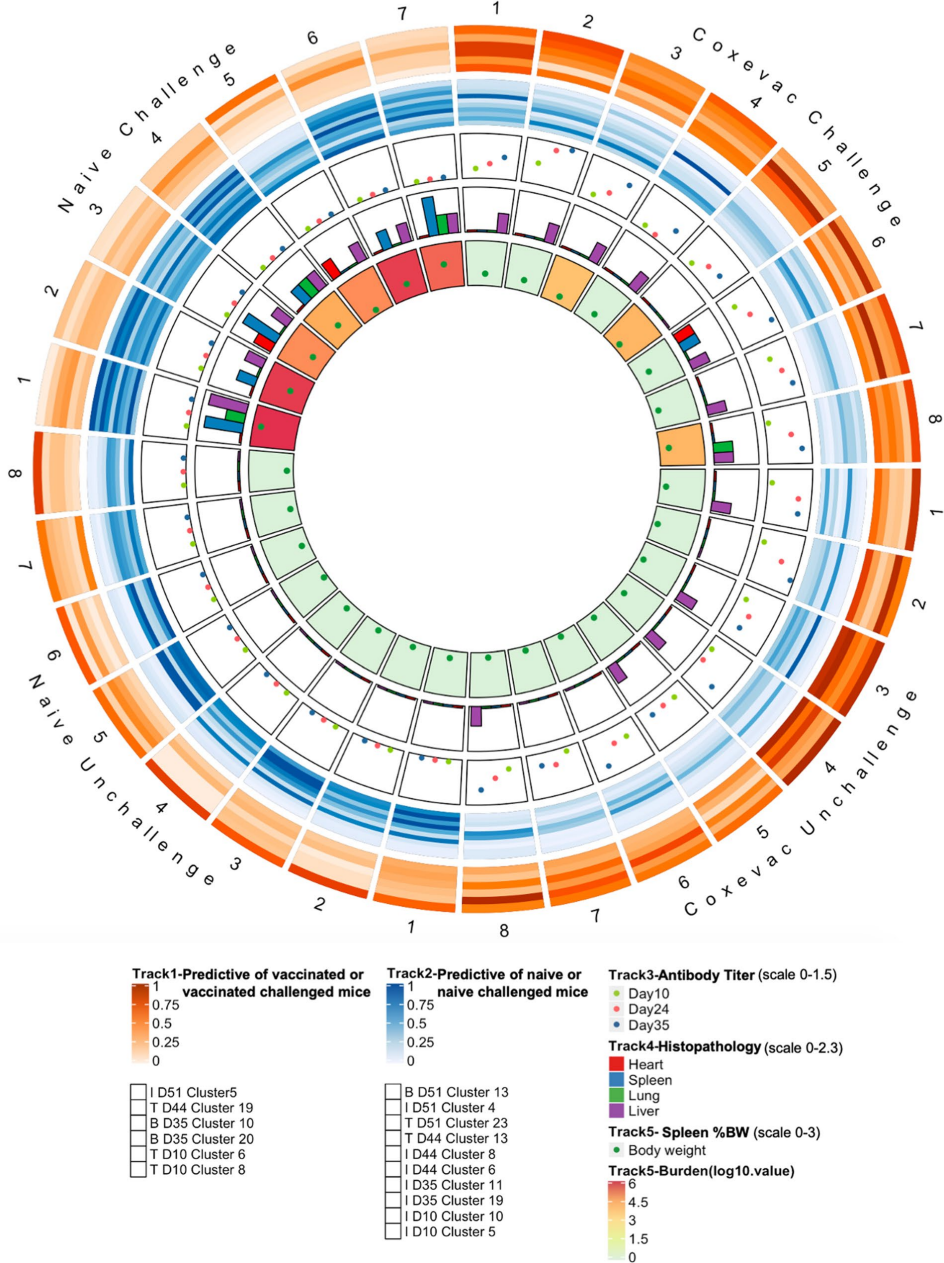
Manual assessment of expression of key markers following *Cb* vaccination and challenge. Manually gated populations were used to determine the frequency of parent for Ly6C+, T-bet+, or CD73+ cells in major immune populations from tgHLA-DR3 mice. Heatmaps illustrate the median frequency of parent within each group of mice (n = 8) on Days 10 and 35 post-vaccination, and Days 2 and 10 post- challenge (study Days 44 and 51). The color-coded scale reflects the frequency of parent for each heatmap. The Kruskal–Wallis test for non-parametric data was used to identify statistically significant changes based on pairwise comparisons between treatment groups within each timepoint; the p-value for significant changes are indicated on the graph.

A Circos plot was generated to visually summarize key data from all aspects of the experiment, while retaining information for individual mice (Fig. 8)^35^. The distinct profiles of naïve and vaccinated groups are readily distinguished, as is the impact of challenge to further separate treatment groups. Features of naïve challenged mice are particularly evident at Day 51, as are features of vaccinated challenged mice, such as the abundance of mature NK cells that are shared with the unchallenged naïve and vaccine groups. Other features of vaccination persist from 10 days post-vaccination until 2 days post-challenge, demonstrating the long-lasting impact of vaccination, even in the face of challenge with *Cb*. Pre-challenge naïve and vaccinated mice are largely distinguished by differential abundance monocyte/macrophage populations, mature NK cells, and activated Th1 CD4 and CD8 T-cell populations. Following challenge, vaccinated and naïve mice exhibit distinct distributions of activated naïve CD4 T-cells, Th1 polarized.

CD4 effector memory T-cells, and activated central memory CD8 cells, as well as subsets of monocytes and activated macrophages. Interestingly, the Circos plot also illustrates a naïve challenged mouse (#5) with reduced bacterial burden and splenomegaly with an immune profile similar to vaccinated challenged mice on Day 51.

## Discussion

We report a longitudinal study of *Cb* vaccination and challenge in a transgenic humanized murine model and describe for the first time the dynamics of all major immune cell populations in the response to *Cb.* Detailed profiling of circulating immune cells by mass cytometry, together with discrete measures of vaccination and infection with *Cb*, were used to identify novel hallmarks of the immune response to *Cb.* We developed an analysis workflow to meet the challenge of batch effects and interrogate data from two replicate studies. Subsequent statistical analysis delineated immune populations, both individually and in combination, that distinguish naïve from vaccinated mice through the course of the study. Further analysis determined the correlation of immune populations to measures of vaccination and challenge. Together, these data reinforce the interconnected dynamics of the immune system, highlight cellular populations and markers that are important drivers of the immune response to *Cb,* and will inform evaluation of future candidate vaccines*.*

Mass cytometry enabled detailed analysis of T-cell, B-cell, and innate (NK cell, granulocyte, and monocyte/macrophage) populations in small blood volumes (< 200 μL) from mice under BSL3 containment, and metal-conjugated antibodies were amenable to the extensive fixation necessary to inactivate *Cb*. Technical considerations of working with small blood volumes under BSL3 containment required that samples were processed and antibody-labeled upon collection, rather than banked for simultaneous analysis of all study samples. Consequently, batch effects prevented simultaneous computational analysis of data across timepoints and replicate experiments, thereby limiting statistical power. Several batch correction algorithms have been described for CyTOF data; however, these tools were not sufficient to enable simultaneous clustering-based analysis of the data from this study, or they require additional controls not included in our study^36–38^. To analyze the multiple timepoints from two independent replicate experiments, we developed a novel workflow that leverages established clustering and statistical analysis methods^32,33,39,40^. The strategy computationally defined cell clusters from each timepoint separately for each replicate experiment and then matched clusters between replicate experiments by phenotype. The resulting matched clusters retain source identity from each mouse as well as information for each individual cell. The matched clusters from all timepoints were organized hierarchically, on the basis of their expression profiles, to identify phenotypic classes and provide longitudinal context. Subsequent statistical analysis enabled identification of populations associated with vaccination status as well as correlation to serological data and measures of infection.

Our results suggest a potential role for Ly6C+ naïve T-cells soon after initial exposure to *Cb,* through either vaccination or challenge. We observed multiple post-exposure CD4 and CD8 T-cell populations distinguished by increased expression of Ly6C, a GPI-anchored glycoprotein expressed by hematopoietic derived cells^41,42^. Typically, Ly6C is used to distinguish subsets of murine myeloid populations. In peripheral CD4+ and CD8+ T-cells, Ly6C expression is indicative of engagement with cognate antigen and enhanced proliferation and effector function^43–47^. Ly6C is associated with T-cell activation in the context of intracellular pathogens, most often viruses, and adoption of a Th1 phenotype by CD4+ T-cells^43,44,48^. CD4+ Ly6C+ T-cells are reported to produce elevated levels of cytokines, including IL-27 and IFNγ. In CD8+ T-cells, Ly6C+ cells may identify a subset of memory cells and its expression may facilitate homing to lymphoid tissues^49,50^. While Ly6C lacks a sequence-based homologue in human immune cells, studies of human and murine monocytes suggest that CD14 may be a functional homologue^51^, although expression of CD14 by T lymphocytes remains an open question.

Somewhat surprisingly, neither CD25 nor CD69, two additional markers of T-cell activation, were identified by our analysis as features of T-cell populations that distinguish the treatment groups. This raises the question regarding the role of Ly6C in the context of *Cb* infection and the mechanism(s) by which it impacts T-cell function, as well as for other markers of T-cell functional status in the context of *Cb* infection (e.g. KLRG1, CXCR3).

One of the most notable features of the overall response was the expression pattern of T-bet, a transcription factor canonically associated with Th1 type immune responses. Computational analysis revealed that distinct populations of T-bet+ CD4, CD8, and innate cells distinguish challenge responses in vaccinated and naïve mice, suggesting a multi-faceted role for T-bet in response to *Cb*. Indeed, a report published as this manuscript was in preparation demonstrated that T-bet deficient mice exhibited significant differences from CD4-deficient mice in a vaccine and challenge study, indicating a role for T-bet beyond Th-1 responses^29^. T-bet influences both innate and adaptive immunity by governing the maintenance of maturation in NK cells, enhancing T-cell function and IFNγ production in response to viruses and protozoa, and aiding in B-cell activation to promote viral clearance^52–55^. Expression of T-bet in CD8+ T-cells induces IFNγ production, which can in turn lead to increased T-bet expression in B-cells and promote antibody class switching^53,55,56^. Indeed, we observed T-bet expression not only in T-cells, but also B-cell and innate populations, including NK cells. Post-challenge, increased expression of T-bet in B-cells, which likely promotes class switching to IgG2a, was detected in vaccinated tgHLA-DR3 mice^57^. This pattern of change is generally consistent with a Th1 response to an intracellular pathogen. The broad role we describe here for T-bet in the in vivo response against *Cb* is in contrast to previous reports indicating that *TBX21*, the gene for T-bet, is downregulated by *Cb* during in vitro granuloma formation^58^*.* These divergent observations likely result from differences in human PBMCs stimulated and cultured in vitro^58,^ as compared to circulating immune cells obtained from mice following vaccination and challenge.

CD8 and NK cell populations expressing CD73 were enriched in vaccinated mice following both vaccination and challenge. CD73 and CD39 are ectonucleotidases that convert extracellular ATP to adenosine, a suppressor of inflammatory function^59–63^. In T-cells, CD39 and CD73 promote adhesion and co-stimulation while curtailing effector function, and naïve CD8+ T-cells downregulate CD73 expression following activation^64,65^. In the context of *Cb*, we observed expression of CD73 on naïve CD8 T-cells in vaccinated mice on Day 10. However, on both Day 10 and Day 35 post-vaccination, CD73+ CD44+ CD62L+ CD8 T-cells (central memory) are enriched in vaccinated mice, which may reflect previously naïve CD8 cells that are in transition towards memory and helping to suppress inflammation following vaccination.

There are additional considerations for interpretation of data generated in this study. The small blood volumes available in a longitudinal study and BSL3 containment prevented inclusion of ex vivo stimulation and allowed only for an abbreviated incubation with a Golgi secretion inhibitor. This likely limited the detection of cytokines, including IFNγ, an established integral facet of the response to *Cb*^18,26,66^. The tgHLA-DR3 mice in this study are on the BL/6 background, which exhibit less severe infection and mortality than some other strains^67^. In this regard, BL/6 background mice may more closely approximate the acute non-lethal disease observed in humans.

This study identified multi-faceted roles for multiple population markers and delineated minimal sets of cell clusters to distinguish treatment groups as well as vaccinated mice that controlled *Cb* efficiently versus those that did not. In particular, the observed importance of T-bet provides evidence supporting a Th1-biased immune response to *Cb*. Similarly, the capacity of T-cell subsets to delineate naïve and vaccinated mice after 10 days suggests a robust early T-cell response to vaccination. The profile of the immune alterations following Coxevac vaccination and subsequent challenge detail the response to an effective, albeit potentially problematic, vaccine. Future candidate vaccines that aim to provide effective protection while minimizing reactogenicity will likely need to recapitulate canonical features of effective vaccination (IFNγ and antibody production) along with facets of the alterations in immune cell populations including participation of monocytes, depletion of circulating mature NK cells, and the involvement of both naïve and memory T-cell populations.

## Materials and methods

### Mice

Female wild-type BL/6 mice, NCI A/JCr (AJ) and Balb/c mice (6–8 weeks old) to assess inactivation of *Cb* were obtained from Jackson Labs (Bar Harbor, ME, US). Female mice transgenic for HLA-DR3 (5–11 weeks old) were obtained under commercial license^68^. Mice were maintained under BSL3 conditions in microisolator cages (Smart Flow, Tecniplast, Westchester, PA, USA) at the Regional Biocontainment Laboratory, Colorado State University, Fort Collins, CO, USA. Animals were provided water and rodent chow ad libitum and evaluated daily to detect changes in body weight, body condition, behavior, and activity level. All studies were performed in accordance with all institutional guidelines and regulations under a protocol approved by the Colorado State University Institutional Animal Care Committee (16–6,844) and following approval by the Animal Care and Use Review Office (ACURO).

### *Coxiella* strains

A stock of *Coxiella burnetii (Cb)* Nine Mile strain was prepared using axenic culture conditions. Propagation was as described^69^ using acidified citrate cysteine medium (ACCM-2) pH 4.75 (Sunrise Science Products, San Diego, CA, USA) seeding flasks with 10^6^ genome equivalents (GE) of *Cb* Nine Mile strain phase 1 RSA 411 (BEI Resources, Manassas, VA, USA). Flasks were incubated for nine days on a shaker (75 rpm) at 37 °C in 2.5% O_2_, 5% CO_2_ environment. Bacteria were recovered by centrifugation (18,000×*g*, 30 min), resuspended in sucrose phosphate buffer, and stored at – 80 °C^70^. The GE/mL of the stock was determined to be 2.17 × 10^7^ GE/µL by quantitative polymerase chain (qPCR) using the LSI VetMax *Cb* absolute quantification kit (Life Technologies, Lissieu, France) and Light Cycler 480 (Roche Diagnostics, Indianapolis, IN, USA.). Infectivity was initially confirmed by i.p. injection into NCI A/JCr mice and was further assessment by i.p. injection into (4 × 10^4^ and 4 × 10^5^ genome equivalents) BALB/c mice. For both A/JCr and BALB/c mice, infection was measured on Day 10 post infection and confirmed by presence of splenomegaly and quantification of bacterial burden by qPCR.

### Determination of the protective dose of Coxevac

A single lot (0101EG1A) of Coxevac (360 mL) was provided by Ceva Animal Health (Lenexa, KS), and received by CSU under a USDA import permit. Coxevac is a formalin inactivated phase I *Cb* corpuscular antigen formulation preserved with thimerosal. Coxevac commercial concentration is in arbitrary “Q fever” antigenic units relative to a proprietary standard. A BCA protein assay (Pierce Biotechnology, Rockford, IL, USA) determined a protein concentration of 86 µg/mL, and qPCR measured 1.3 × 10^6^ GE/mL. To determine the protective dose of Coxevac, BL/6 mice (n = 5 per dose, 14 weeks old) were inoculated subcutaneously (s.c.) with saline containing serially diluted Coxevac (0, 0.4, 2, and 10 µg). At Day 42 post-vaccination, mice were challenged i.n. with 1 × 10^5^ GE of *Cb*. Mice were sacrificed on Day 52 (10 days post-challenge), and heart, liver, lung, and spleen were harvested. Histological lesions were scored on a graded scale of 0–4 (0 = no lesions; 1 = minimal, < 5% affected; 2 = mild, 5–10% affected; 3 = moderate, 10–25% affected; 4 = severe, > 25% affected). The splenic bacterial burden was determined by qPCR and spleen/body weight ratios were calculated.

### Vaccination with Coxevac and subsequent challenge with live *Cb*

Two sets of 16 tgHLA-DR3 mice (5–11 weeks old) were randomly assigned to two and four groups respectively. Mice were vaccinated s.c. with 10 µg (100 µL) of Coxevac at Day 0; mice in control groups received phosphate buffered saline (PBS) (100 µL). At Day 42, mice were chemically restrained (ketamine, 100 mg/kg and xylazine, 10 mg/kg, intraperitoneal (i.p.) for inoculation with *Cb* by i.n. route with a target dose of 10^5^ genome equivalents, as determined by qPCR, in 20 µL. The control group (n = 5) received PBS (20 µL). Whole blood samples were collected from the submandibular region on Days 10, 24, 35, 44 and 51 and the resulting serum was used for serological analysis. The white blood cells from 10, 35, 44, and 51 cells were analyzed by CyTOF. All mice were euthanized at Day 52 and spleens were collected and weighed. Tissues (heart, lung, liver, spleen) were collected in 10% formalin for paraffin embedding and stained with hematoxylin and eosin for histopathological review. Histological lesions were scored on a graded scale of 0–4 (0 = no lesions; 1 = minimal, < 5% affected; 2 = mild, 5–10% affected; 3 = moderate, 10–25% affected; 4 = severe, > 25% affected).

### Inactivation of *Cb* in infected samples

To allow for analysis outside the BSL3 facility, a sample inactivation procedure was established to ensure that fixed blood samples from infected mice were safe for transport and for subsequent immune marker assessment by CyTOF. Fixation in 4% paraformaldehyde for 1 h was determined sufficient to eliminate infectivity in spleen cell suspensions used to inoculate A/J mice, which are highly permissive for *Cb* infection^67^. For each timepoint, barcoded and labeled leukocyte samples from infected mice were incubated in 4% formaldehyde PBS solution for two hours to inactivate any infectious *Cb* and stored frozen. Due to limited blood volumes, to confirm *Cb* inactivation for each timepoint, a representative splenic sample from an infected mouse that had been previously aliquoted and stored following disassociation by serial passage through 70 mm and 40 mm filters was inactivated in parallel with the blood and stored in the same manner. The parallel inactivation at each timepoint of the banked *Cb* infected spleen sample provided a common control across experiments to confirm *Cb* inactivation and enable blood sample release from the BSL3 facility. To confirm inactivation, the representative splenic sample was injected i.p. into naïve susceptible NCI A/JCr mice and on Day 10 post-inoculation with inactivated materials the spleens were recovered from the A/JCr mice. Inactivation was confirmed by assessing splenomegaly and bacterial burden as detected by qPCR.

### Murine blood collection

A solution of 169 mM EDTA, prepared from 500 mM EDTA (Thermo Fisher) diluted in Milli-Q water, was used to pre-coat 200 μL capillary tubes. At each timepoint, 150–200 μL of whole blood was collected per mouse by submandibular bleed into the capillary tubes. Blood samples from each mouse were transferred to a 1.7 mL conical tube containing 22 μL of RMPI and 0.5 mg/mL brefeldin A and incubated at room temperature for 45 min prior to red blood cell (RBC) lysis and antibody labelling of leukocytes.

### Serology

Plasma was processed for serology as described previously^71^. Whole blood was collected as described above. A 10 μL aliquot of plasma was recovered after centrifugation (300 g for 3 min) and stored at -80C. Anti-*Cb* antibody levels in the plasma were determined by ELISA (Q Fever Ab Test, IDEXX Laboratories, Westbrook, ME, USA). The secondary antibody was replaced with peroxidase conjugated protein A/G (Pierce Biotechnology, Rockford, IL, USA) or goat anti-mouse IgG (H + L) (Jackson ImmunoResearch Laboratories, Rockford, IL, USA). ELISA measurements were reported as absorbance values measured at 450 nm (OD_450_) and baseline corrected, using plasma from naïve animals.

### Mass cytometry

Whole blood collected at each timepoint was depleted of erythrocytes using the eBioscience 10X multispecies RBC lysis buffer. Following lysis, samples were barcoded, surface labelled with antibody, and fixed prior to storage at − 80 °C until release following *Cb* inactivation confirmation. Subsequently samples were thawed, labelled intracellularly, and analyzed by mass cytometry (see Supplementary Material).

### Data analysis

Following acquisition of mass cytometry data, the resulting files were processed in accordance with previously reported methods^72–74^. For each sample, data were normalized using bead standards to account for machine variance, using proprietary software from Fluidigm. Viable single cells, T-cells, B-cells, and innate cells were identified by manual gating with FlowJo 10 software (Supplementary Fig. 3a). Values from mass cytometer channels containing non-biological data were removed and the resulting FCS files used for downstream analysis. Statistical tests were performed as indicated in the figure legends using GraphPad Prism V8, unless otherwise noted. Additional details provided in supplementary methods.

### Clustering analysis and cluster matching

First, the data from each replicate experiment were considered independently. For each timepoint the data were manually divided into T-cell, B-cell, and innate cell populations (Supplementary Fig. 3). Then resulting populations from each timepoint were clustered independently to identify the distinct subpopulations of cells present. Cell clusters were defined based on the abundance of the markers detected on each cell, grouping together cells with highly similar profiles using the Vortex implementation of the X-shift algorithm, a k-nearest neighbors method^32^, according to the labels as indicated in Fig. 2 and in Supplementary Information. Cluster analysis used between 3.4e5 and 1.2e6 cells per timepoint from each experiment. Subsequently, the differential abundance of each cluster between treatment groups was determined for individual timepoints by pairwise t tests and visualized using SPADEVizR^39^ (Supplementary Fig. 6).

To identify phenotypically similar clusters conserved between two cognate replicate timepoints, cluster profiles from the replicate experiments were compared using Pearson’s correlation. The list of putative pairings was ranked according to correlation value. Clusters with a frequency of less than 0.02% of input cells were removed from the analysis and a lower limit of r = 0.6 was used to limit the number of candidate pairings. For each cluster, starting with the highest correlation value, candidate pairings were manually evaluated with the basis of phenotype (cluster profile), overall distribution between treatment groups, and cluster size. In some instances, candidate pairings with good phenotypic correlation and similar abundance did not have similar distributions between experimental groups; however, this did not preclude acceptance as a matched pair. As a result of this analytical approach, clusters with no corresponding match in the replicate experiment or with a frequency of less than 0.02% of total input were excluded from further analysis.

### Heatmaps, logistic regressions, correlation matrix, discriminant analysis, and t-SNE

Details related to the production of heatmaps, Elastic Net logistic regression, correlation matrix calculation, t-SNE map generation, and linear discriminant analysis are provided in Supplementary Material. Briefly, heatmaps and the correlation matrix were both created used the R packages Pheatmap, using the Ward.D2 method for hierarchical clustering, Pvclust for bootstrap analysis, and rcorr function for Spearmann correlation analysis^75,76^. NbClust was used to determine the appropriate segmentation parameters^77^. Elastic Net analysis of phenotypic classes and individual clusters, as well as multi-mode classification to identify the minimal set of highly predictive clusters and linear discriminant comparison of challenged mice, was performed using the R package caret^78^. Detailed methods provided in Supplementary Material.

### Circos plot

The details of how the Circos plot was constructed is detailed in the Supplementary data section^35^. The Circos plot was constructed with the R package Circlize^79^ and was structured to include 31 sections, representing each mouse included throughout the duration of the study: 8 sections each for Coxevac unchallenged, Coxevac challenged and Naive unchallenged, and 7 sections for Naive challenged. For each timepoint, data from the two clusters with the highest positive or negative correlation coefficient to vaccinated or vaccinated challenged mice were used. The heatmap mode was utilized to represent the correlation coefficient with the value for each cluster rescaled to 0–1. Similarly, antibody titer, histopathology score, spleen %bw, and bacterial burden were integrated into the Circos plot. Scatterplot mode was used to show the value of antibody and body weight, while the histogram mode was used to indicate the pathology. A gradient color scheme was applied to reflect the value of bacteria burden.

## Supplementary information

Supplementary information 1

Supplementary information 2

Supplementary information 3

## Data Availability

Raw data, processed data, and source code for reproduction of the results are publicly available via https://flowrepository.org/id/FR-FCM-Z2LH and https://github.com/reeves-lab.
