# Supplementary material for: Novel multiparameter correlates of Coxiella burnetii infection and vaccination identified by longitudinal deep immune profiling
Source: Sci Rep. 2020 Aug 7;10:13311. doi: 10.1038/s41598-020-69327-x (PMC7414860; doi:10.1038/s41598-020-69327-x)
Supplement: Supplementary file 3 — Supplementary information 3 [file 41598_2020_69327_MOESM3_ESM.docx]

Novel multiparameter correlates of *Coxiella burnetii* infection and vaccination identified by longitudinal deep immune profiling

P. M. Reeves^1*^, S. Raju Paul^1^, L. Baeten^2^, S. E. Korek^1^, Y. Yi^1^, J. Hess^1^, D. Sobell^1^, A. Scholzen^3^, A. Garritsen^3^, A. S. De Groot^4,5^, L. Moise^4^, T. Brauns^1^, R. Bowen^2^, A. E. Sluder^1^, M. C. Poznansky^1*^

**Supplementary Materials**

-Supplementary Figures

-Supplementary Results

-Supplementary Methods

-Supplementary References

**
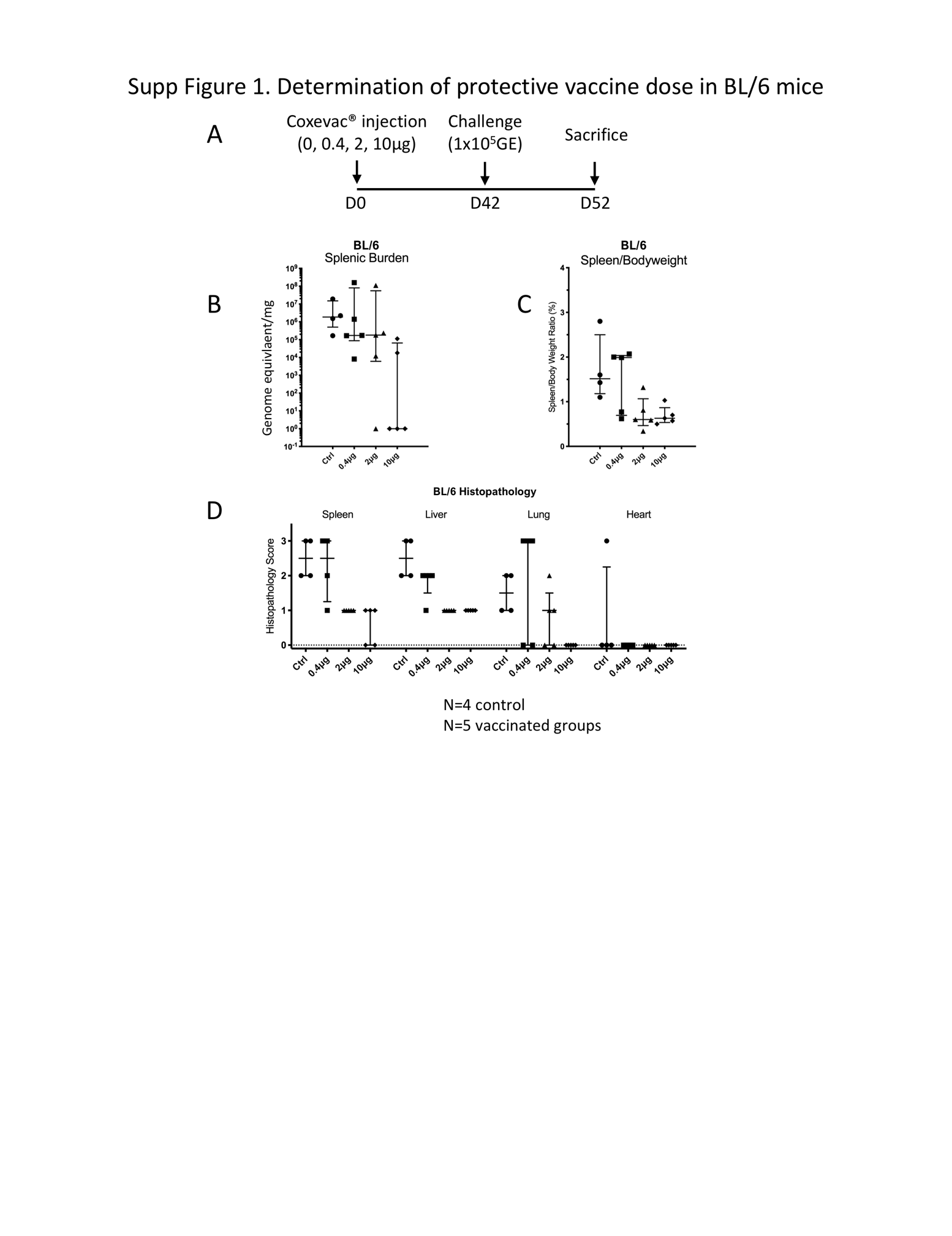
**

**Supplementary Figure 1. Determination of protective vaccine dose in BL/6 mice**

(A) 8-week old BL/6 mice (controls N=4, vaccinated groups N=5 each) were injected subcutaneously with indicated quantities of Coxevac. After 42 weeks, mice were infected i.n. with a standardized axenic stock of *Cb* Nine Mile containing the indicted number of genome equivalents GE. The optimal vaccine dose was calculated based on total number of mice in each group that displayed signs of protection against infection based on either (B) splenic bacterial burden (GE/mg spleen determined by qPCR), (C) spleen:body weight ratio (%BW) or (D) histopathology. Histological scoring of observed lesions: 0= no lesions; 1= minimal, < 5% affected; 2= mild, 5-10% affected; 3= moderate, 10-25% affected; 4= severe, > 25% affected).


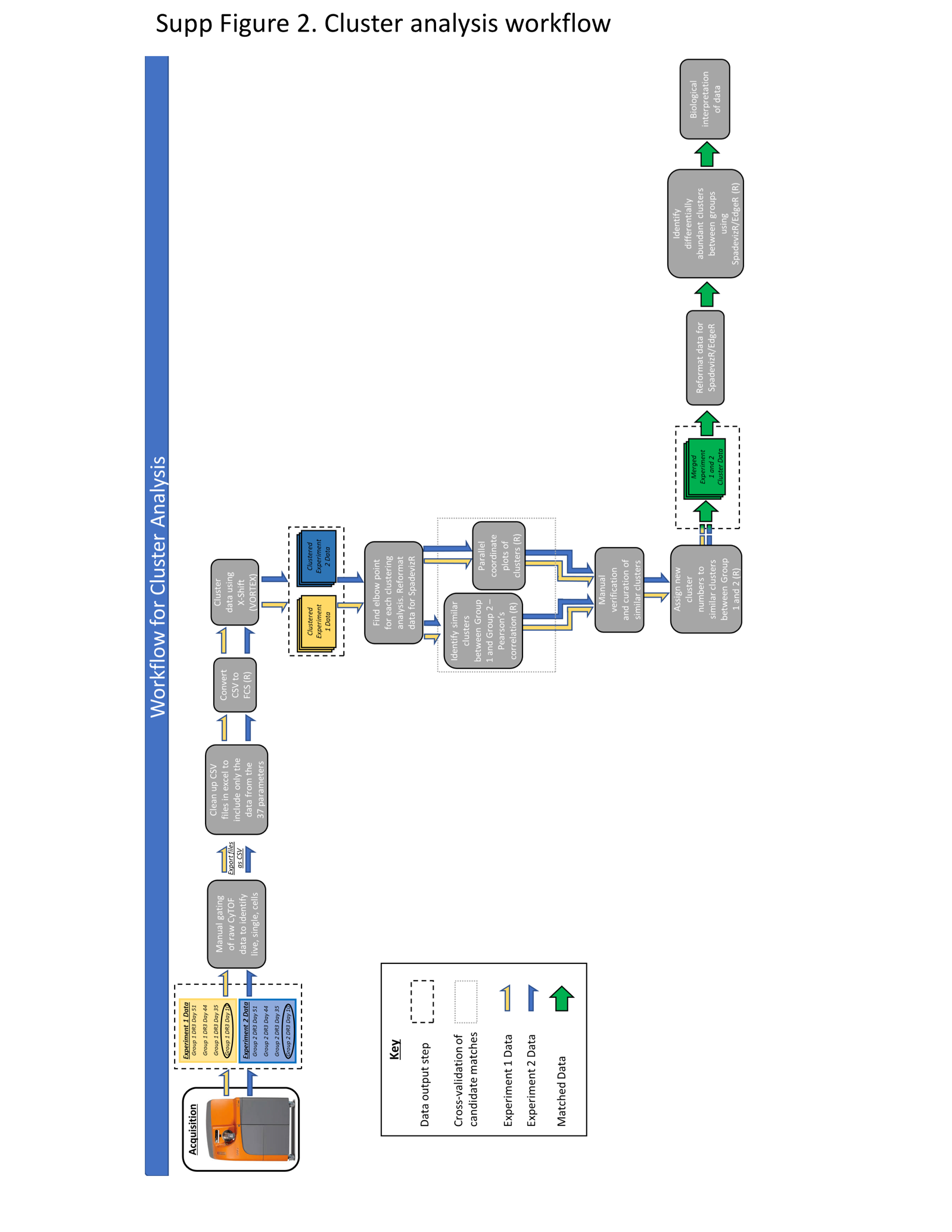


**Supplementary Figure 2. Cluster analysis workflow**

Flowchart depicting the workflow for analysis of CyTOF from replicate experiments using clustering and subsequent statistical and correlative identification of features enriched in experimental groups.


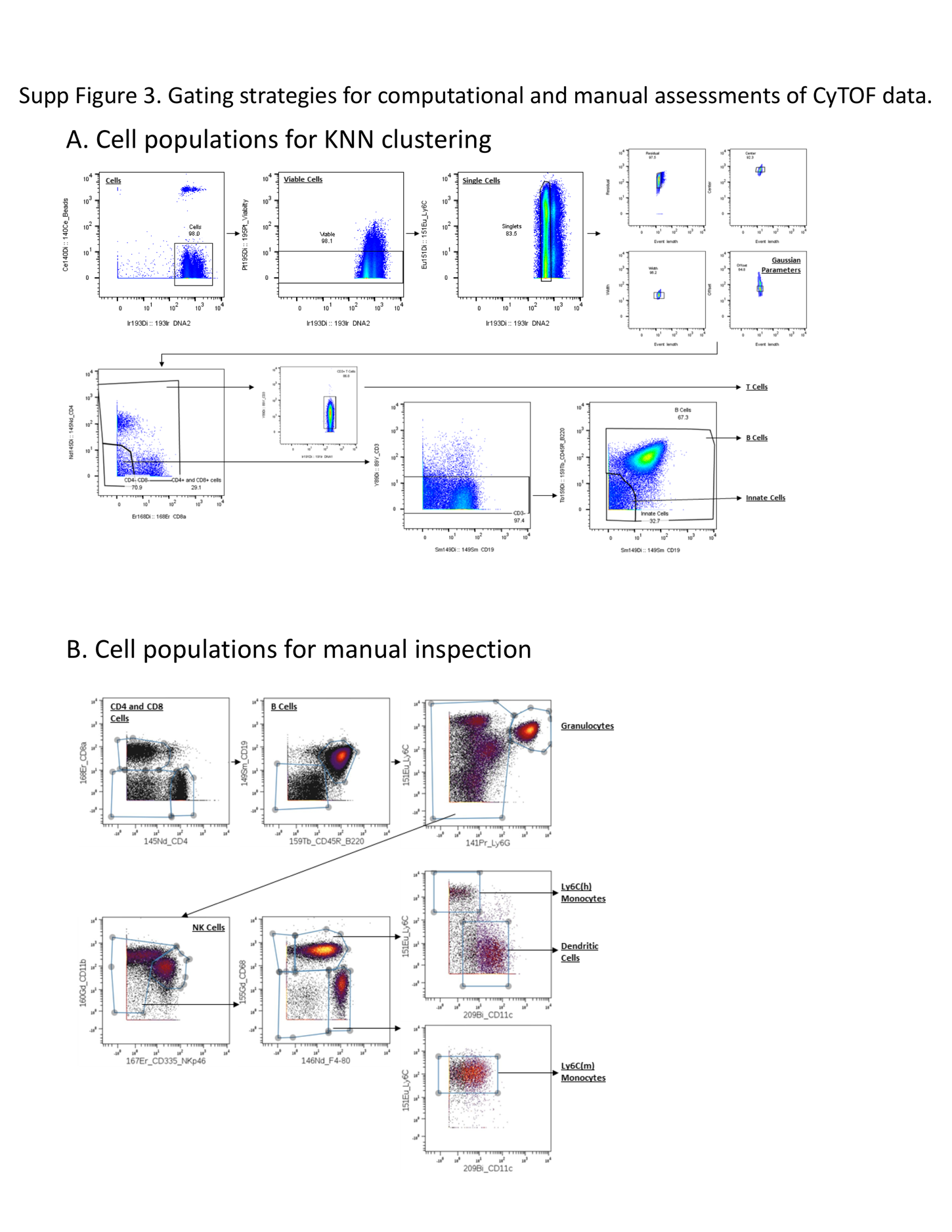


**Supplementary Figure 3. Gating strategies for computational and manual assessments of CyTOF data**

(A) CyTOF data were gated to identify viable single cells, and then T cell, B cell and Innate cell populations for use in cluster analysis. (B) Viable single cells identified as in A were manually gated to identify major cell subpopulations – T cells, B cells, Granulocytes, NK cells, Ly6C(h) monocytes, Ly6C(m) monocytes and dendritic cells.


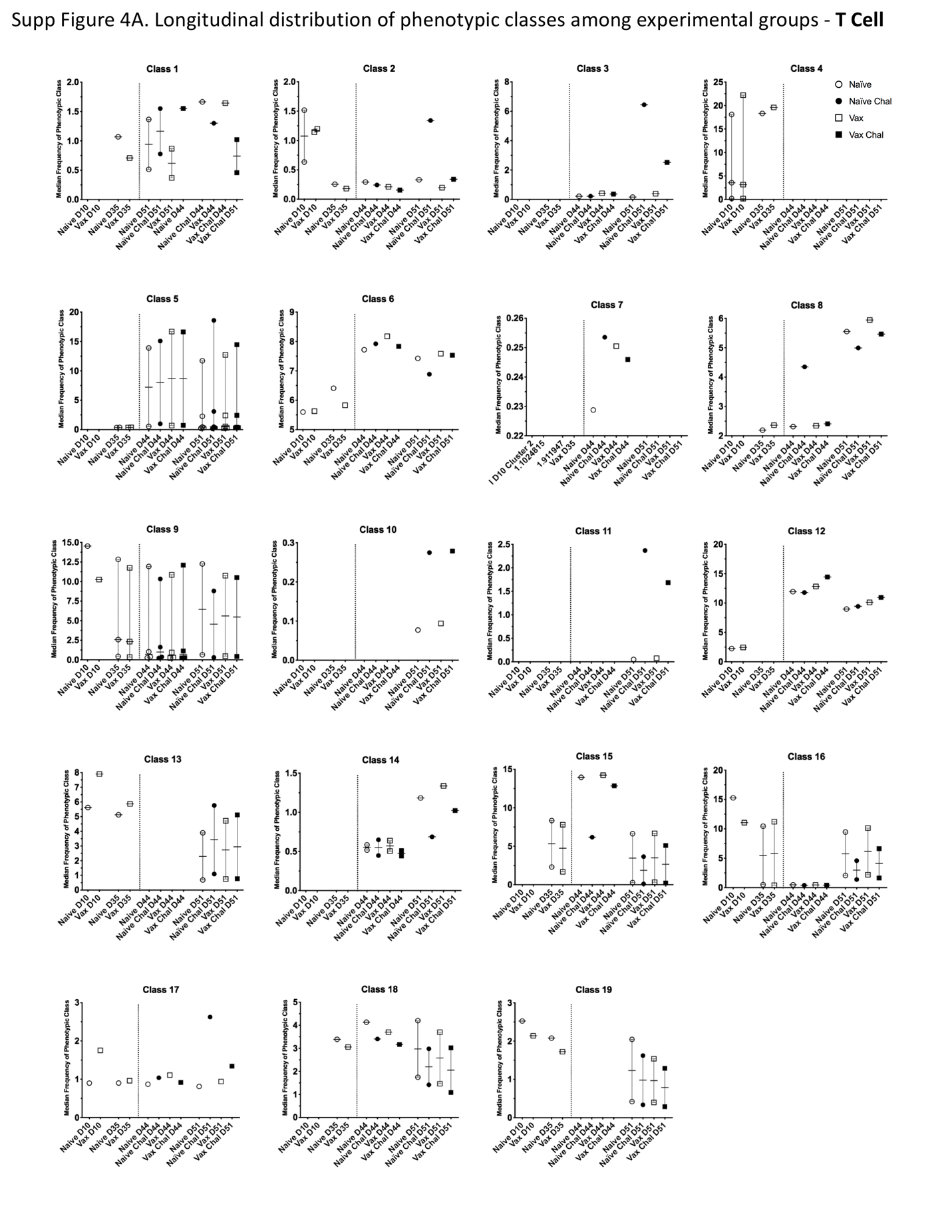


**
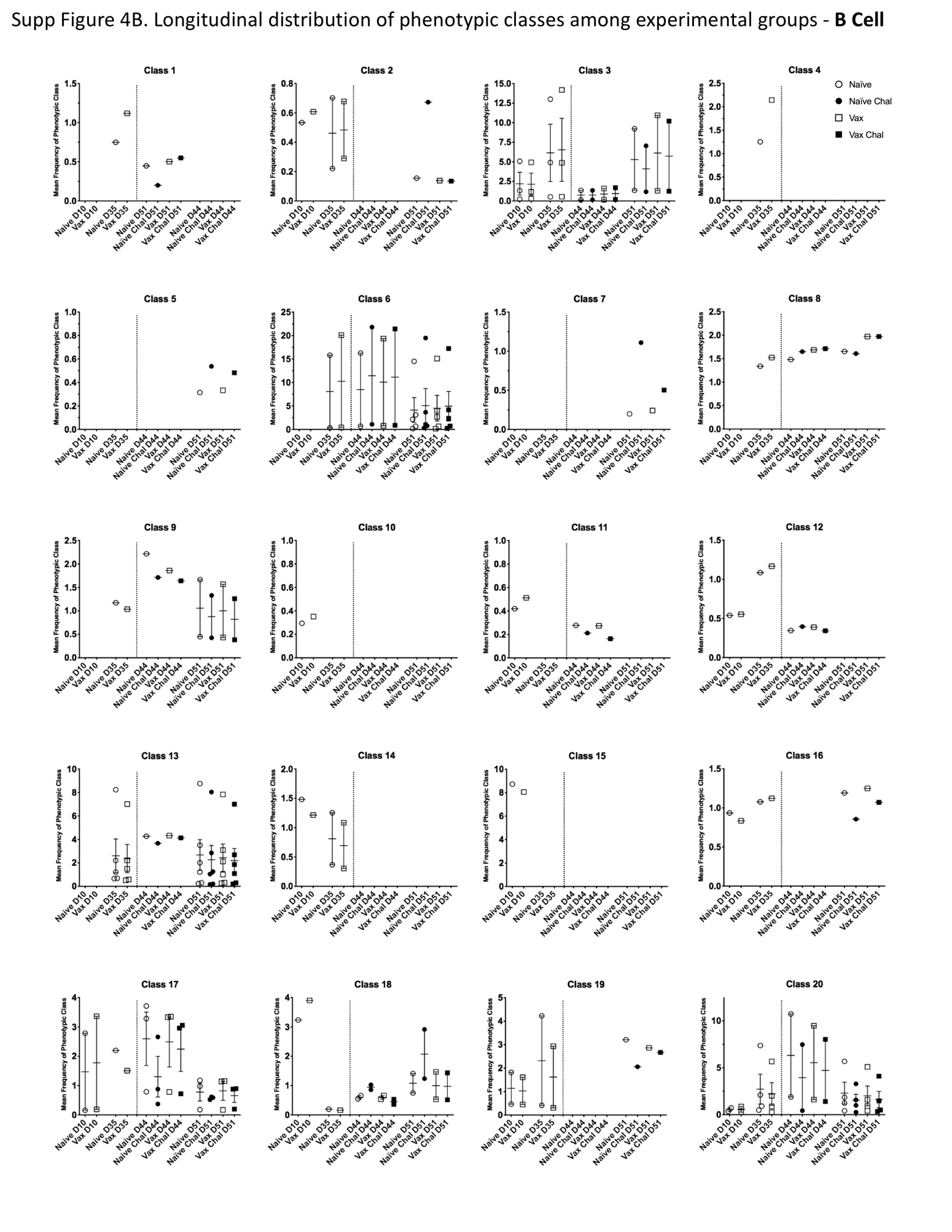

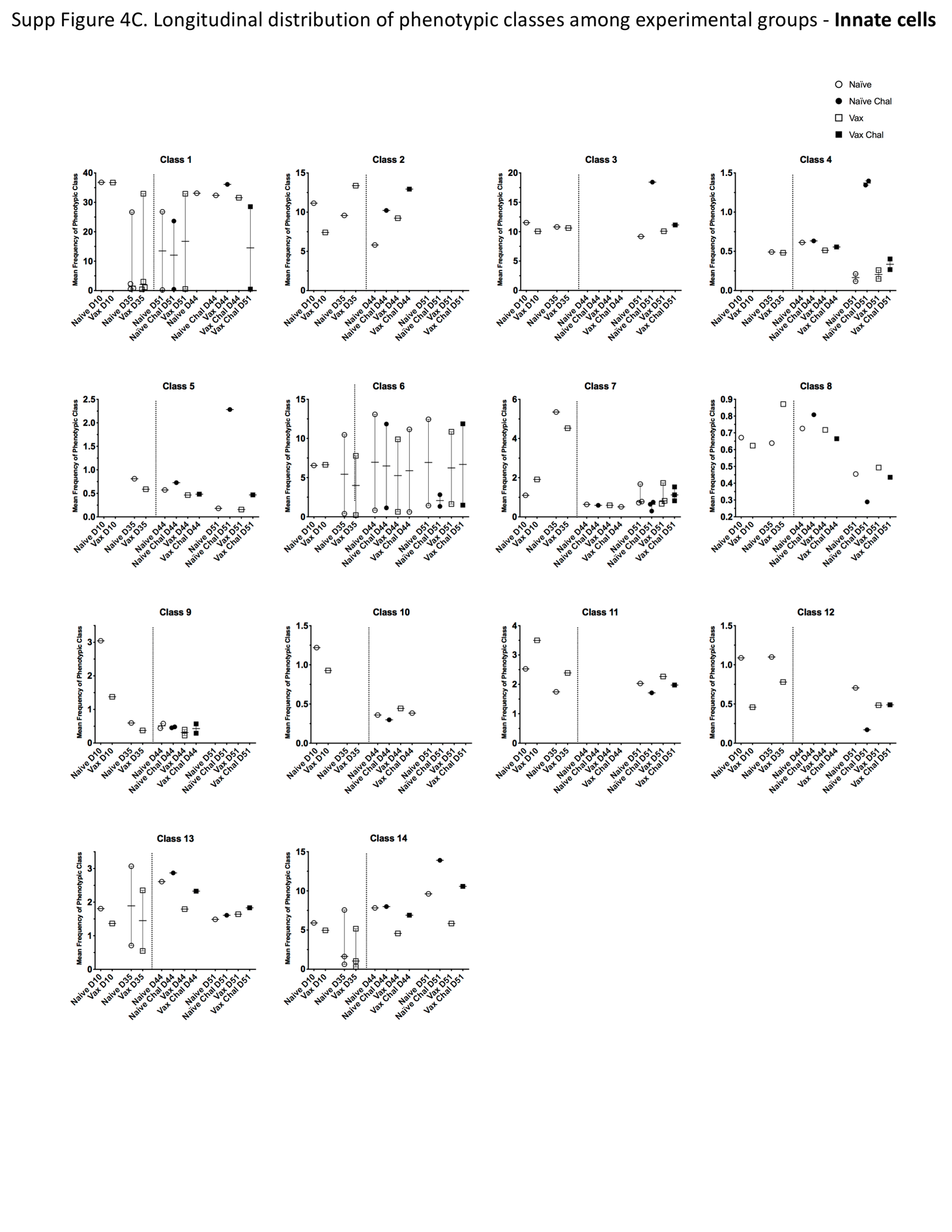
**

**Supplementary Figure 4. Longitudinal distribution of phenotypic classes among experimental groups.** The mean frequency of each phenotypic class is reported per day across groups, both post-vaccination and post-challenge, for T cells (A), B Cells (B), and innate populations (C). The dotted vertical line denotes the distinction between pre- and post-challenge timepoints.


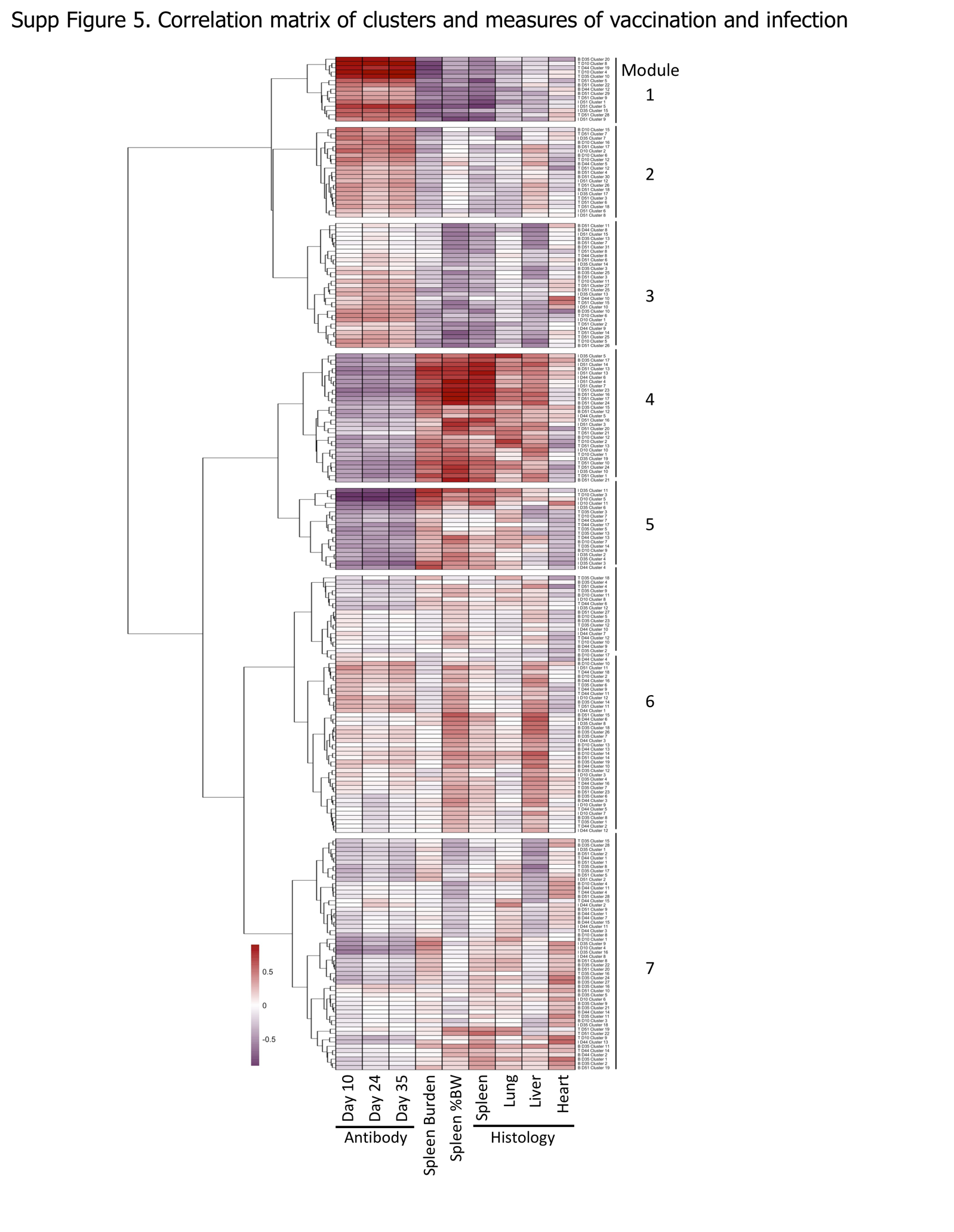


**Supplementary Figure 5. Correlation matrix of clusters and measures of vaccination and infection**.

The heatmap depicts positive (red) and negative (blue) correlation values for each matched cluster from T cells, B cells and Innate cells across all timepoints, with respect to antibody titer, spleen bacterial burden, spleen %BW, and histopathology scores. Hierarchical clustering was performed using an R implementation of the Ward.D2 minimum variance method. The number of modules for segmentation (7 modules, also in Fig 4A) was determined by unsupervised analysis with NbClust and segmentation was performed on the basis of dendrogram tree height.


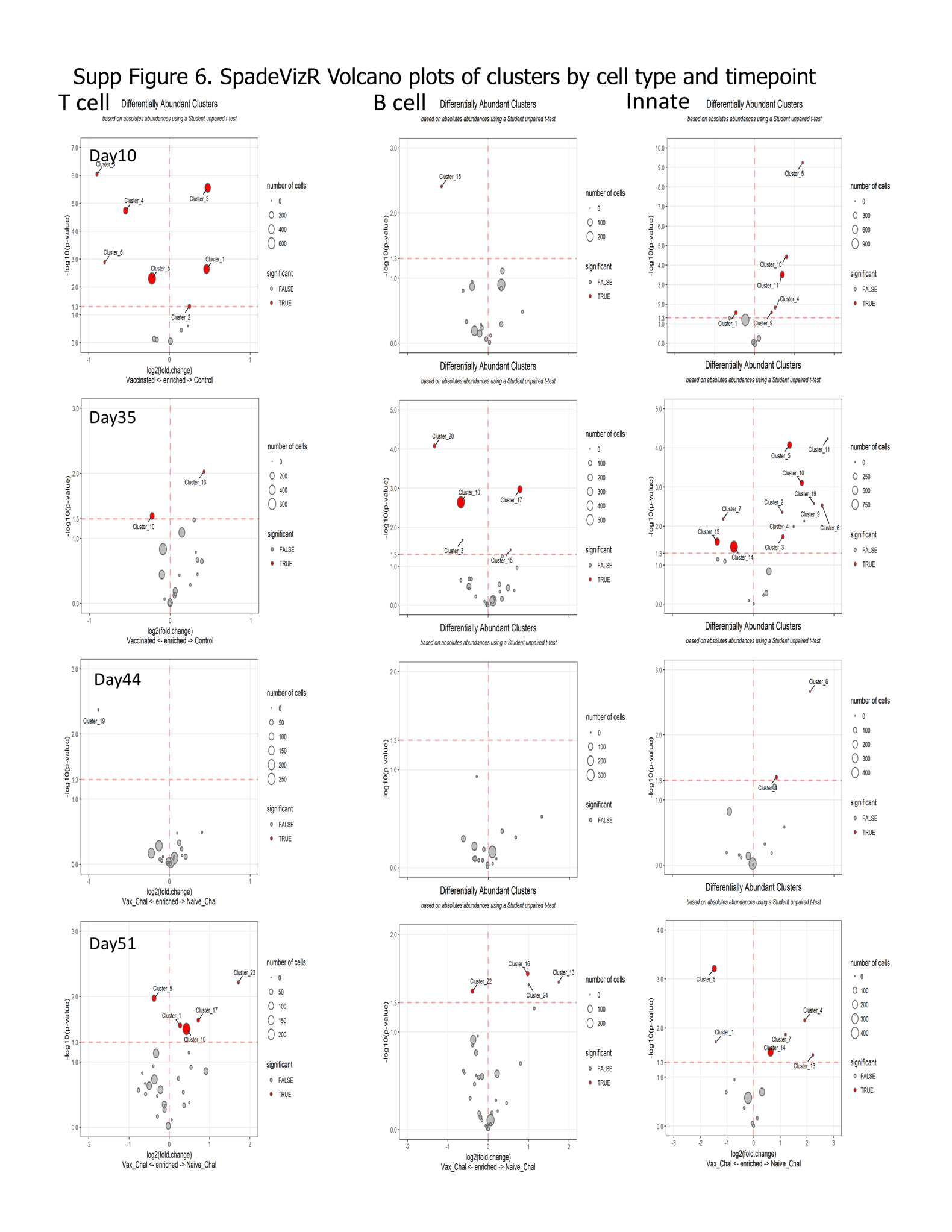


**Supplementary Figure 6. SpadeVizR volcano plots by cell type and timepoint.**

Volcano plots of T cells, B cells, and Innate Cells from day 10, day 35, day 44, day 51. The y-axis is the -log of the p-value and the x-axis is the log2(fold change). Vaccinated or vaccinated challenged mice are on the left portion of the graph and naïve or naïve challenged mice are on the right portion of the graph.


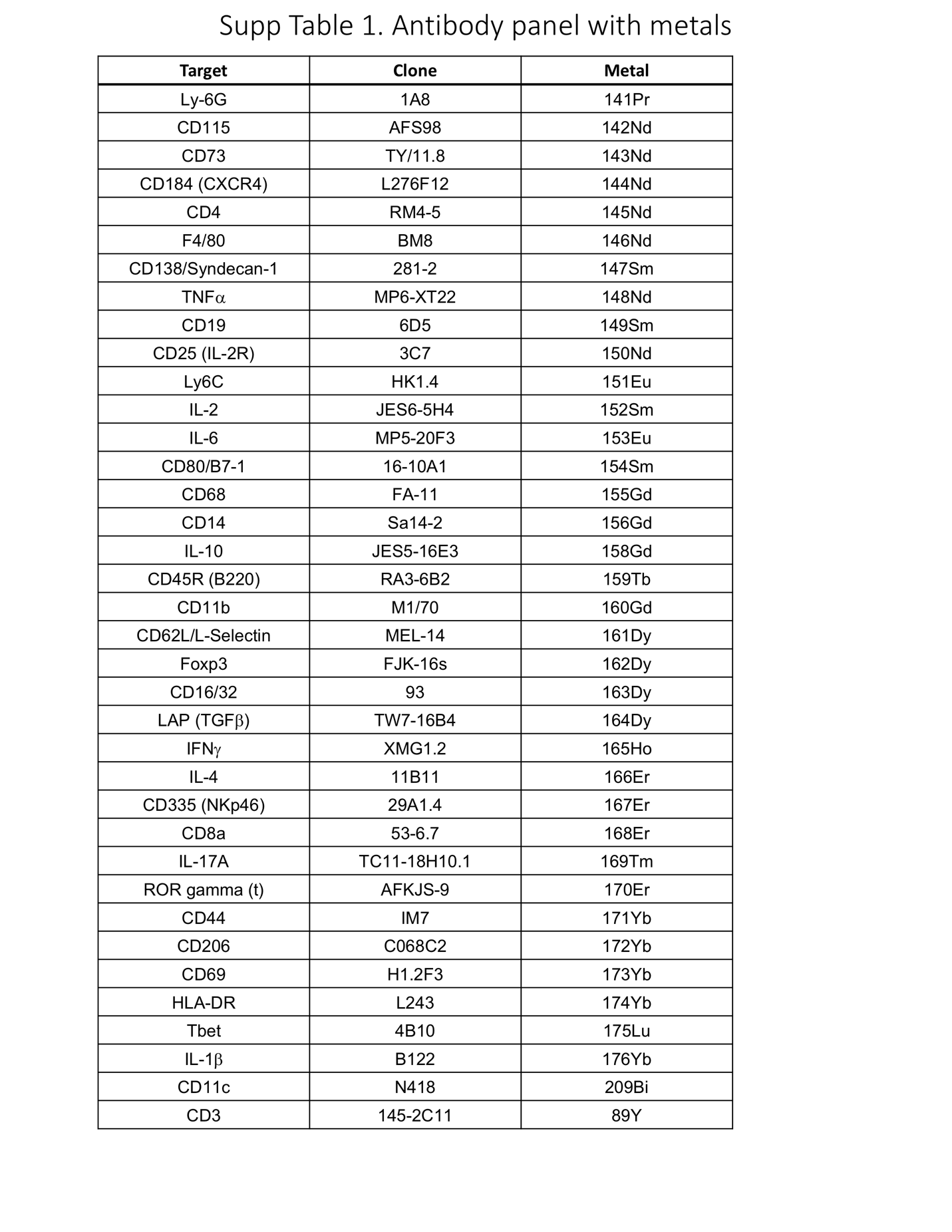


**Supplementary Table 1. Antibody panel.**

Antibody targets, clones, and conjugates are listed for the panel used in this study

**
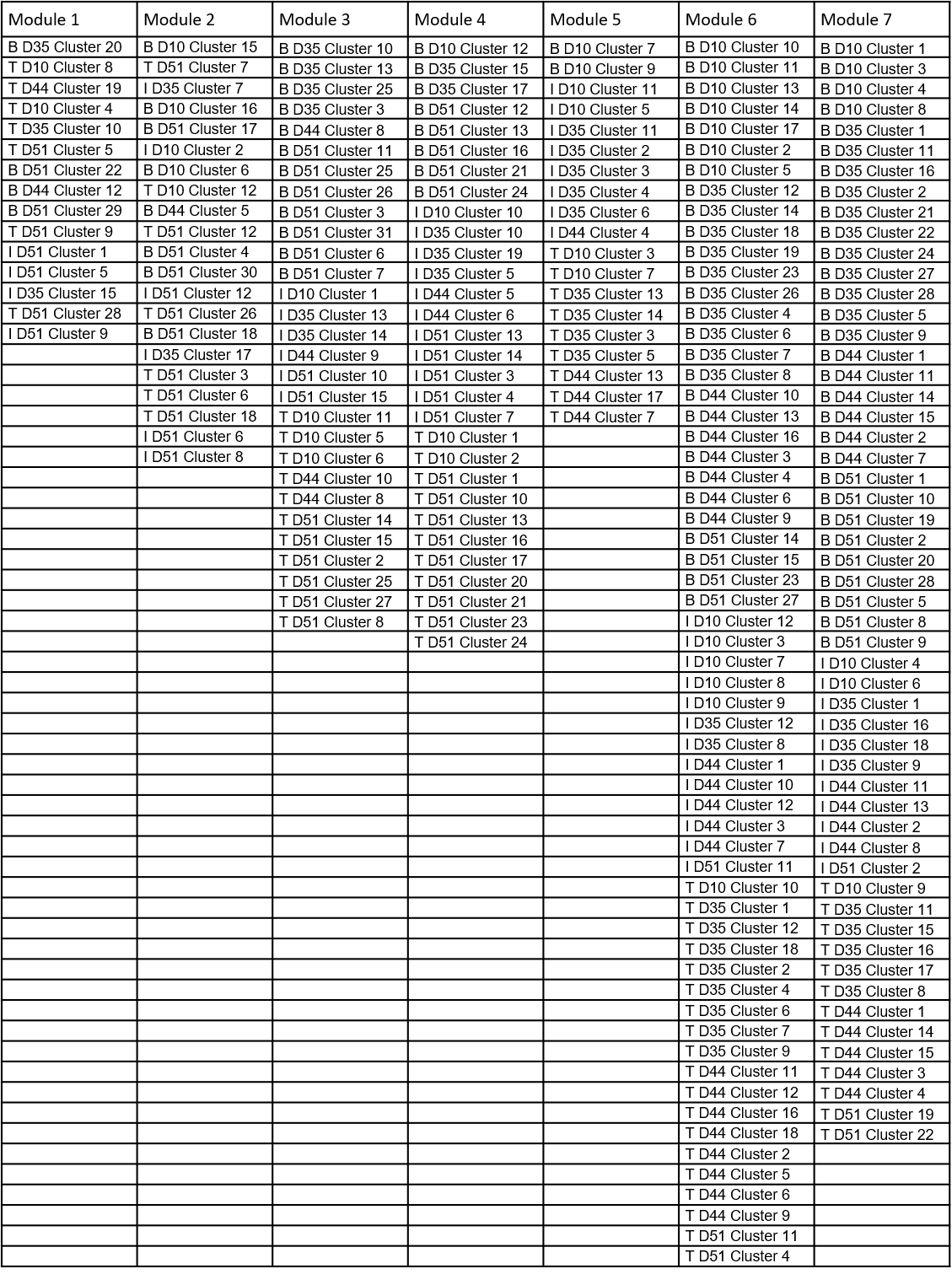
**

Supp Table 2. Module assignments for clusters (from Fig 4)

**Supplementary Table 2. Module assignments for clusters (from Fig 4)**. List of clusters from in each module derived from the correlation matrix and mapped to the t-SNE.


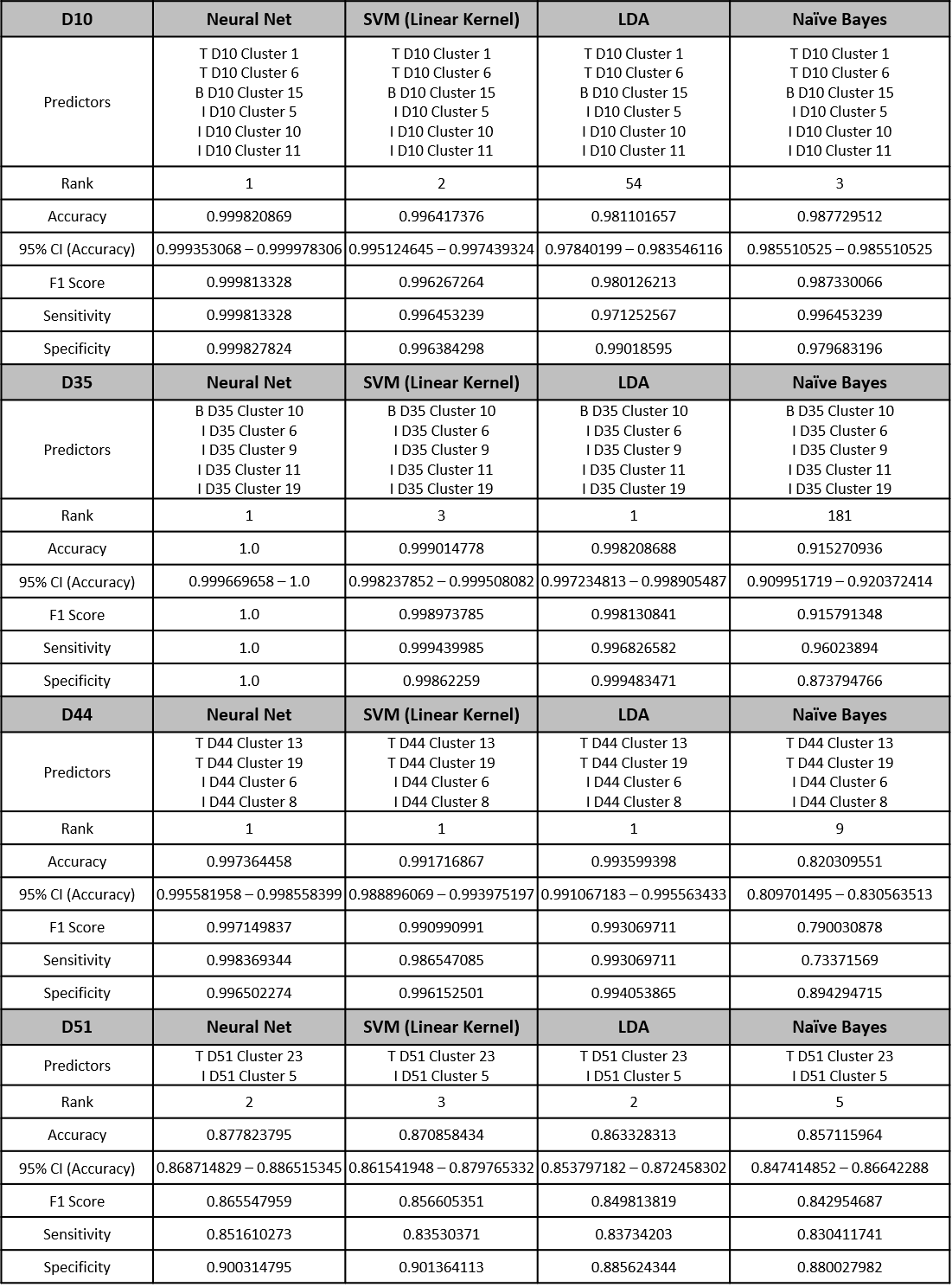


Supp Table 3. Selection of Predictive Population Combinations

**Supplementary Table 3. Selection of Predictive Population Combinations**

Identification of population combinations with greatest predictive power to distinguish naïve from vaccinated mice or naïve challenged from vaccinated challenged mice. For each timepoint a series of iterative computational tests using the Caret R package were performed to identify the minimal combination of matched clusters with the highest predictive value, specificity and accuracy. Four computational tools, Neural Net, Support Vector Machine (SVM), Linear Discriminant Analysis (LDA), and Naïve Bayes were employed to evaluate all possible combinations of clusters. The best predictive combination of clusters (predictors) and the respective performance measures are provided for each day and method. Presented are the combinations that contain at least one cluster associated with naïve mice and one with vaccinated mice.

**Supplementary Results**

**Link of phenotypic families to experimental group across timepoints**

For each timepoint, the capacity of individual phenotypic families to predict group assignment was assess by EN regression analysis and was used to identify key populations through the course of the study (Fig 3). The overall distribution of T-cell, B-cell, and innate cell populations between experimental groups over the duration of the study was considered in addition to the day-by-day breakdowns.

Seven T cell classes (four CD4+ and three CD8+) were predictive of group assignment (classes T2, T3, T4, T13, T16, T17). Expression of Ly6C was a consistent feature shared by five of the seven T cell classes and CD73 characterized three of the seven classes. Class T3, comprised of CD4+ CD44+ Ly6C+ TGFβ+ T-bet+ cells, had the greatest predictive value and was the lone class identified by EN for vaccinated challenged mice (Day 44). By day 10 post-challenge, two CD4+ classes (T2 and T3) and one CD8+ class (T17) were enriched in naïve challenged mice as compared to vaccinated challenged mice, though only class T2 was selected by EN.

Four classes of B cells were identified as predictive by EN (B2, B4, B6 and B18). Notably, only two classes of B cells predictive of vaccinated mice were detected, both on day 35 (B4, B6). Both classes share similar phenotypes (CD19+ CD45R+ CD44+ CD62L+, HLA-DR+), consistent with antigen presenting memory cells^1,2^. However, class B4 had higher predictive value and was differentiated by expression of CD115, a marker usually associated with macrophage and osteoclast differentiation with reported expression in splenic plasmablast cells. Classes B2 and B18 were distinct from B4 and B6 in their association with naïve and naïve challenged mice as well as the absence of CD62L and expression of Ly6C, which may indicate plasma cell like status^3^.

Within innate immune cells, 9 of the 14 classes had predictive associations with treatment groups (I2, I4, I5, I6, I8, I9, I11, I12, I14). Within the six classes sharing a CD335- CD206+ CD11b+ phenotype, I5 and I6 are both CD11c+, with Class I5 distinguished by expression of HLA-DR. Interestingly, on day 51, 10 days post-challenge, both classes contain clusters identified by EN, with I5 (D51 Cluster 4) associated with naïve mice, and Class I6 (D51 Cluster 5) linked to vaccinated challenged mice. HLA-DR expression on cells in naïve challenged mice likely reflects an ongoing inflammatory state, where continued antigen presentation would be advantageous ^4^. Similarly, classes I2 and I4 are linked to naïve and naïve challenged mice respectively and share similar phenotypes that differ on the basis of Ly6G (I2) and HLA-DR (I4). The CD206+ Ly6C+ CD68+ CD11c+ cells in class I8 were detected at each timepoint and were linked to vaccinated mice on Day 35. In contrast, CD206+ Ly6C+ Class I9 lacked CD11b thereby indicating a myeloid cell lineage, and was linked to naïve mice on days 10, 35, and 44. Class I11 was notable for expression of the canonical monocyte marker CD16 along with CD11b and its link to vaccinated mice at both pre-challenge timepoints. Two additional classes, I12 and I14, associated with naïve mice prior to challenge, expressed CD11b+, CD11c+, the NK marker CD335 as well as T-bet, which promotes maintenance of a mature NK phenotype. Increased circulating mature NK cells in naïve mice, as compared with vaccinated mice, likely results from the recruitment of NK cells to the site of vaccination with inactivated whole-cell *Cb* ^5^.

The relationships and dynamics of particular cell sub-populations and markers through the course of vaccination and challenge become apparent through this analysis. Interestingly, the predictive value of some populations is greatest when they first emerge as opposed to when they are the most abundant, suggestive of the importance of timing and dynamics in shaping a robust immune response to *Cb*.

**Predictive cells clusters to distinguish vaccinated from naïve mice**

For each timepoints following vaccination the capacity of individual clusters to predict assignment to naïve or vaccinated groups was determined by elastic net (EN). An analogous assessment was conducted between naïve challenged and vaccinated challenged groups for the two post-challenge timepoints.

On day 10 post-vaccination, the vaccinated group was distinguished from naïve mice by three T cell clusters (D10 clusters T8, T6, T4), including two CD8+ clusters expressing CD73, Ly6C, CD62L and a CD4+ Ly6C+ CD62L+ cluster, as well as a CD19+ CD20+ CD44+ HLA-DR+ B-cell cluster (D10 cluster B15) (Fig 5A). Interestingly, three innate immune cell populations and two T-cell populations (D10 clusters I5, I10, I11, T3, T1) distinguished naïve mice. The innate immune populations included T-bet+ CD44+ CD11b+ NK cells and CD44+ CD73+ monocytes, while the T cells included memory CD4+ T cells and CD8+ T cells expressing CD73, but not Ly6C.

EN analysis of day 35 post-vaccination identified an increased number of clusters that distinguished treatment groups (Fig 5B). Those clusters most strongly associated with vaccinated mice included the D35 T, B, and innate cell populations B20, B10, I13, I14 and T10. Both B cell clusters are CD19+ B220+ CD44+ CD62L+. The innate clusters both expressed CD11b and CD44, with one expressing CD16 and the other expressing IL-1b, IL-6, and CD73 in addition to a number of population markers (Ly6G, Ly6C, CD68, CD44 and CD206). Among those clusters distinguishing naïve mice on day 35, the most prominent were the D35 innate and B cell populations I11, I19, I5, I9, I2, I6, I10, B17, and B15. These populations encompassed T-bet+ CD73 monocytes, four T-bet+ NK populations with variable expression of CD11b, CD11c, CD44, and CD62L, as well two CD19+ CD20+ CD44+ B cell populations.

At two days post-challenge (day 44), a CD4 T-cell population expressing Ly6C, T-bet, CD206, and TGFβ (D44 cluster T19) was the single cluster associated with vaccinated challenged mice as compared to naïve challenged mice (Fig 5C). Naïve mice were distinguished by an individual innate CD73+ T-bet+ CD206+ TGFβ+ monocyte population (D44 cluster I6). By 10 days post-challenge (day 51, Fig 5D), a single innate immune population expressing CD115, CD206, CD11b, CD11c, F4/80 and CD68 (D51 cluster I5) was strongly associated with vaccinated challenged mice. Two clusters, including HLA-DR CD11b CD11c-expressing monocytes and Ly6C+ immature CD4+ T cells, were associated with naïve challenged mice (D51 clusters I-4 and T23).

Through the course of these analyses, a clear trend of increasing immune diversity following vaccination emerges. However, challenge with live *Cb* winnows the number of predictive features found in circulation and highlights the role for T-cells, NK and monocyte/macrophage populations during the initial 10 days following challenge. Further analysis of lung, spleen and other organs and later timepoints would likely reveal additional alterations that would further inform the overall status of the immune response in naïve and vaccinated animals, both after vaccination and challenge.

**Supplementary Methods**

**Blood sample preparation and antibody labelling.**

Samples were prepared as described previously, with some modifications^6^. Specifically, whole blood (up to 200 μL) was incubated with 4 mL of isotonic RBC lysis buffer at room temperature for 10 min, after which the samples were washed twice with cell staining buffer (CSB; made with 0.5% bovine serum albumin and 0.02% sodium azide in isotonic PBS). To identify live cells, the sample was incubated with 100 μL of 2.5 μM cisplatin (Fluidigm) for 10 min at 4°C. Unbound cisplatin was removed by washing the cells with CSB. For barcoding, each sample was incubated in 500 μL of 1X Fix Buffer (Fluidigm) for 10 min at room temperature, followed by washing cells with 1X perm buffer (Fluidigm), and labeled with using a 20-plex barcoding kit (Fluidigm). Post barcoding, samples were washed twice with CSB, pooled, and incubated in CSB containing antibody at 4°C for one hr. Samples were then washed twice with CSB and fixed for 2 hrs with freshly prepared 4% formaldehyde solution PBS to inactivate *Cb*. Post fixation, samples were frozen at -80°C and stored until released following inactivation confirmation.

Frozen samples were thawed in a water bath at 37°C and incubated in 1 mL of eBioscience FoxP3 transcription factor buffer at room temperature for 30 min, washed twice with eBioscience permeabilization buffer, and incubated in isotonic perm buffer containing antibodies against intracellular antigens at 4°C for 45 min, washed twice with permeabilization buffer and incubated in 4% formaldehyde PBS solution for 10 minutes. After washing with CSB, samples were incubated with 1mL of 0.125μM Iridium based intercalator (Fluidigm) at room temperature for 20 min, and then washed once with CSB. Immediately prior to analysis by mass cytometry, samples were washed twice in Milli-Q water containing four element calibration beads (Fluidigm) at a 1:10 dilution, filtered through a 40μm filter, and diluted to 1x10^6^ cells/ml. For each acquisition, a sample not labeled with antibody was prepared to control for possible contamination from reagents. Samples were acquired at a rate of approximately 550 events per second on a Helios mass cytometer (Fluidigm). All antibody clones, concentrations, and tags are provided in Supplementary Table 1.

**Data analysis**

**Clustering Analysis** The parameters for clustering, elbow points, input number of cells, Pearson’s and Spearmann’s correlation values, and other details are contained in the supplementary file “Clustering Summary.xlsx”. All clustering was performed with Vortex, which uses a weighted K-nearest neighbor density estimation (KNN-DE) to compute the density estimate for each data point within a dataset and searches for the local density maxima in a nearest-neighbor graph to identify cluster centroids^7^. The remaining data points are then connected to the centroids via density-ascending paths in the graph, forming clusters. Vortex then searches for density minima on a straight path between neighbor centroids and merges them where necessary. This ensures that neighboring clusters, with similar phenotypic profiles, are in fact distinct clusters. Lastly, Vortex uses a fixed Mahalanobis distance threshold to merge clusters. The algorithm generates clusters for multiple K values and suggests a K value on the basis of an elbow point calculation. In this study, each cell population described above was clustered separately, with independent computations for each timepoint from both experiments.

Once an appropriate K value was identified, information on the phenotypic profiles corresponding to each cell cluster, retaining per mouse assignments, were exported from Vortex. To increase the statistical power of the experiment, we identified phenotypic clusters that were present in replicate experiments at each timepoint, using Pearson’s correlation. This allowed us to combine both experiments, report only reproducible data and hence increase the statistical power of the analysis. Differentially abundant clusters across experimental groups at each timepoint were determined by pair-wise t tests using SPADEVizR^8^, a R package for statistical analysis and visualization.

**Phenotypic Classes**

To construct the heatmap matched clusters, were organized by hierarchical clustering using Euclidean distance measure and the Ward.D2 method using the Pheatmap R package and Hclust function on the basis of marker expression, excluding values for IFNg, CD14, and IL-17A^9^. Segmentation into phenotypic classes used the cut-tree height function of Pheatmap and was based on the number of modules suggested through unsupervised analysis using NbClust and the Ward.D2 method^10^. The structure of hierarchical clustering derived dendrogram was confirmed through a 1000 iteration bootstrapping procedure using pvclust package.

**Correlation analysis**

The correlation matrix of cell clusters and measures of antibody titer, bacterial burden, and histopathological scoring was determined using Spearmann correlation option of the Rcorr package. The resulting correlation values for each cluster were then used to construct heatmap using Pheatmap and hierarchically clustered using the Euclidean distance and Ward.D2 method. The same correlation values were also used to construct a t-SNE map using the RtSNE package, thereby reducing the dimensionality of the matrix^11,12^. Segmentation of the correlation matrix into modules was informed by the optimal number of clusters determined through unsupervised analysis with NbClust using the Ward.D2 method. Subsequently, the matrix was segmented on the basis of dendrogram height using the cut-tree function of Pheatmap.

**Elastic Net Analysis**

Elastic net analyses of both phenotypic classes and individual clusters were performed for each timepoint individually using the Caret package, with the cross-validation metric set to optimize accuracy of group classification. The median abundance of each phenotypic class for each individual mouse was used while retaining group assignment, with each variable scaled to have a mean of 0 and a standard deviation of 1. Mixing parameters were determined using four-fold repeated cross validation. The elastic net analysis of individual clusters per timepoint was carried out in the same manner, utilizing the frequency of parent (e.g. T cell, B cell, innate cell) for each cluster for each individual mouse.

**Minimal cluster set with maximum predictive value**

The R package Caret was used to implement four models: Neural Net, Support Vector Machine (SVM), Linear Discriminant Analysis, Naïve Bayes Classifier. For each timepoint the frequency of parent (e.g. T cell, B cell, innate cell) for each cluster for each individual mouse was used to determine the optimal subset of parameters for group assignment at each timepoint, we extracted from the elastic net a maximum of ten clusters and exhaustively searched for the set of clusters that maximized classification accuracy across four models. A maximum of ten clusters was chosen at each timepoint, and all possible subsets were determined. The max of 10 clusters was chosen due to computational burden. Excluding the empty set, a set of features of size n has 2^n^-1 possible subsets. Hence, we use a maximum of 1023 subsets across four classification models at each timepoint.

Clusters were ranked according to the magnitude of their logit coefficient produced by the elastic net regression, and a maximum of ten clusters was chosen in descending order. For each unique subset of predictors, a support vector machine with linear kernel, linear discriminant analysis, naïve Bayes classifier, and a single-hidden layer feed-forward neural network were optimized by bootstrap resampling. Input parameters for each model were chosen according to the set that maximized combined classification accuracy on bootstrap hold-out sets across a hyperparameter tuning grid-search.

Performance Metrics**:**  Reported (in the table) are metrics for assessing classification performance across bootstrap hold-out sets. We chose to include, for each day, the set of predictors that gave the best overall classification accuracy across all model. 95% confidence intervals for classification accuracy are calculated by an exact binomial test. The “Rank” of each subset is the rank after ordering all combinations in descending order according to their resamples accuracy. All other calculations were computed using the following confusion matrix and equations:

TP = True Positive

Predicted: Vaccinated/Vaccinated Challenge

Actual: Vaccinated/Vaccinated Challenge

FP = False Positive

Predicted: Vaccinated/Vaccinated Challenge

Actual: Naïve/Naïve Challenge

TN = True Negative

Predicted: Naïve/Naïve Challenge

Actual: Naïve/Naïve Challenge

FN = False Negative

| **Predicted** | Vaccinated/Vaccinated Challenge | Naïve/Naïve Challenge |
| --- | --- | --- |
| Vaccinated/Vaccinated Challenge | TP | FP |
| Naïve/Naïve Challenge | FN | TN |

Predicated: Naïve/Naïve Challenge

Actual: Vaccinated/Vaccinated Challenge

$$precision=\frac{TP}{(TP+FP)}$$

$$sensitivity=\frac{TP}{(TP+FN)}$$

$$specificity=\frac{TN}{(FP+TN)}$$

$$F1 score=\frac{\left( 1+\beta^{2} \right)*precision*sensitiviy}{{((\beta}^{2}*precision)+sensitivity)}$$

**Linear discriminant analysis**

First, individual clusters from across all timepoints were assessed for their respective capacity to distinguish the naïve challenged mice from the 5 vaccinated challenged mice that had no detectable bacterial burden. For each timepoint the abundance of each cluster relative to the total cells per mouse was used to conduct a linear discriminant analysis (LDA) to assess their individual capacity to segregate the two groups. The model corresponding to each cluster was ranked according to bootstrap resampling accuracy. The bootstrap analysis considered 1000 iterations for each cluster. The rankings were used to identify an elbow point where the resampling accuracy decreased precipitously, no models under 42% accuracy were considered. The number of clusters and the accuracy range are as follows

Day 10: 15 clusters range: 99.7-50.9%

Day 35: 15 clusters range: 77.9-54.2%

Day 44: 10 clusters range: 91.1-42.8%

Day 51: 15 clusters range: 83.8-63.1%

These clusters were then used to conduct a second LDA to determine the capacity of all combinations of up to 3 clusters to discriminant between naïve challenged and *Cb*-neg vaccinated challenge mice on a per day basis. Subsequently, the corresponding values from *Cb*-pos vaccinated challenged mice were projected onto the feature space and visualize as a density plot. The corresponding density plots were used to identify cluster sets where the distribution of *Cb*-pos vaccinated challenged mice trended towards naïve challenged mice while also considering the overall discriminant value.

**Supplementary References**

1. Camp, R. L., Kraus, T. A., Birkeland, M. L. & Puré, E. High levels of CD44 expression distinguish virgin from antigen-primed B cells. *J. Exp. Med.* **173**, 763–6 (1991).

2. Roche, P. A. & Furuta, K. The ins and outs of MHC class II-mediated antigen processing and presentation. *Nat. Rev. Immunol.* **15**, 203–16 (2015).

3. Wrammert, J., Källberg, E., Agace, W. W. & Leanderson, T. Ly6C expression differentiates plasma cells from other B cell subsets in mice. *Eur. J. Immunol.* **32**, 97–103 (2002).

4. Porcheray, F. *et al.* Macrophage activation switching: an asset for the resolution of inflammation. *Clin. Exp. Immunol.* **142**, 481–9 (2005).

5. Pradier, A. *et al.* Modulation of T-bet and Eomes during Maturation of Peripheral Blood NK Cells Does Not Depend on Licensing/Educating KIR. *Front. Immunol.* **7**, 299 (2016).

6. Reeves, P. M. *et al.* Application and utility of mass cytometry in vaccine development. *FASEB J.* 1–11 (2017). doi:10.1096/fj.201700325

7. Samusik, N., Good, Z., Spitzer, M. H., Davis, K. L. & Nolan, G. P. Automated mapping of phenotype space with single-cell data. *Nat. Methods* **13**, 493–496 (2016).

8. Gautreau, G. *et al.* SPADEVizR: An R package for visualization, analysis and integration of SPADE results. *Bioinformatics* **33**, 779–781 (2017).

9. Kolde, R. pheatmap: Pretty Heatmaps. (2019).

10. Charrad, M., Ghazzali, N., Boiteau, V. & Niknafs, A. NbClust : An R Package for Determining the Relevant Number of Clusters in a Data Set. *J. Stat. Softw.* **61**, (2014).

11. van der Maaten, L. & Hinton, G. Visualizing Data using {t-SNE}. *J. Mach. Learn. Res.* **9**, 2579–2605 (2008).

12. Krijthe, J. H. Rtsne: T-Distributed Stochastic Neighbor Embedding using a Barnes-Hut Implementation. (2015).
